# Multiplicity of carotene patterns derives from competition between phytoene desaturase diversification and biological environments

**DOI:** 10.1038/s41598-020-77876-4

**Published:** 2020-12-03

**Authors:** Mathieu Fournié, Gilles Truan

**Affiliations:** 1 TBI, Université de Toulouse, CNRS, INRAE, INSA, Toulouse, France; 2 Adisseo France S.A.S., 10 place du Général de Gaulle, 92160 Anthony, France; 3 Groupe Avril, 11 Rue de Monceau, 75378 Paris, Cedex 08, France

**Keywords:** Biochemistry, Enzymes, Isoenzymes

## Abstract

Phytoene desaturases catalyse from two to six desaturation reactions on phytoene, generating a large diversity of molecules that can then be cyclised and produce, depending on the organism, many different carotenoids. We constructed a phylogenetic tree of a subset of phytoene desaturases from the CrtI family for which functional data was available. We expressed in a bacterial system eight codon optimized CrtI enzymes from different clades. Analysis of the phytoene desaturation reactions on crude extracts showed that three CrtI enzymes can catalyse up to six desaturations, forming tetradehydrolycopene. Kinetic data generated using a subset of five purified enzymes demonstrate the existence of characteristic patterns of desaturated molecules associated with various CrtI clades. The kinetic data was also analysed using a classical Michaelis–Menten kinetic model, showing that variations in the reaction rates and binding constants could explain the various carotene patterns observed. Competition between lycopene cyclase and the phytoene desaturases modified the distribution between carotene intermediates when expressed in yeast in the context of the full *β*-carotene production pathway. Our results demonstrate that the desaturation patterns of carotene molecules in various biological environments cannot be fully inferred from phytoene desaturases classification but is governed both by evolutionary-linked variations in the desaturation rates and competition between desaturation and cyclisation steps.

## Introduction

**Figure 1:**
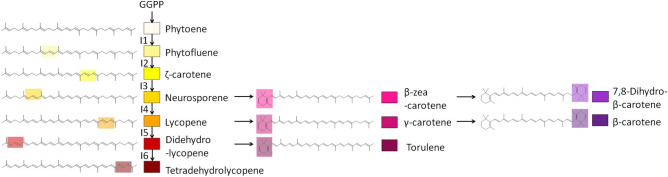
Carotenes-forming enzymatic reactions in fungi and bacteria. Desaturation and cyclisation steps are presented as vertical or horizontal arrows, respectively. The number following the capital I letter in the desaturation reactions corresponds to the supplementary number of double bonds relative to phytoene. The colours associated with the molecules were arbitrarily chosen and are reused throughout the article. The molecular details and absorbance data of the presented molecules are recapitulated in the Supplementary Table S1.

Carotenoids are organic pigments produced by plants, algae, fungi, and bacteria and can be subdivided into two families of molecules, carotenes and their oxidised counterparts, xanthophylls^1^. Carotenes represent a series of tetraterpenes with nine to fifteen double bonds, some being conjugated with no, one, or two rings at their extremities. The number and position of the double-bonds and rings confer specific visible light absorption properties to carotenoids, leading to pleiotropic biological functions. For example, carotenoids can serve as efficient light-harvesting molecules, as well as photoprotectors of light-harvesting complexes^2,3^, and they transfer energy to photosynthetic pigments in purple bacteria^4^ and in other phototrophs. The conjugated double bonds are responsible for the antioxidant properties of carotenoids by limiting the accumulation of singlet oxygen molecules, neutralising free radicals, and preventing potential oxidative damage in non-photosynthetic microorganisms^5^ and fungi^6^ (Fig. 1 and Supplementary Table S1). Industrial interest in carotenoids as food colorants, animals feed, and neutraceuticals has led to a growing interest in producing them from heterologous hosts, in particular microorganisms such as *E. coli* or *S. cerevisiae*^7^.

Carotenoid biosynthesis starts with the symmetrical condensation of two geranylgeranyl pyrophosphate molecules, forming phytoene. A series of successive desaturation reactions convert phytoene into phytofluene, *ζ*-carotene, neurosporene, lycopene (Fig. 1)^8^. These desaturation reactions can be accomplished by a single enzyme (poly-*trans* pathway) or through a cascade of different enzymes (poly-*cis* pathway)^9^. In algae and plants, four different enzymes are necessary to form the final product (all-*trans*-lycopene). The phytoene and the *ζ*-carotene desaturases (PDS and ZDS, respectively) add double bonds in the *cis*-conformation. ZISO (*ζ*-carotene isomerase) and CRTISO (prolycopene isomerase) convert the *cis*-carotenes into di-*cis*-*ζ*-carotene and all-*trans*-lycopene, respectively^10^. Cyanobacteria, as green sulfur bacteria, also possess two desaturases (CrtP and CrtQ) that produce *cis*-carotenes and one isomerase (CrtH) that can transform some of them into the *trans*-configuration^11,12^. *Gloeobacter violaceus* is an exception as it possesses only CrtI instead of the three abovementioned enzymes^13,14^. In fungi and other bacteria, all desaturation and isomerization reactions are catalysed by a single, multifunctional enzyme (CrtI), which directly converts phytoene into various *trans*-desaturated carotenes^15^. By contrast to other phytoene desaturases, CrtI are versatile enzymes classified into four enzymatic subgroups (EC 1.3.99.28, 1.3.99.29, 1.3.99.30, and 1.3.99.31) based on the last product they presumably produce (from *ζ*-carotene to didehydrolycopene)^16–19^. Carotene diversity can be further expanded in later steps with the addition of one or two rings by lycopene cyclases, thereby producing an extensive variety of symmetrical or asymmetrical cyclised carotenes, such as *β*-zeacarotene, dehydro-*β*-carotene, *γ*-carotene, *β*-carotene, and the fungi-specific torulene (Fig. 1 and Supplementary Table S1).

Interestingly, when expressed in heterologous hosts, CrtI enzymes exhibit distinct desaturation patterns^20–22^. For example, the well-studied enzyme CrtI from *Pantoea ananatis*^19^ forms tetradehydrolycopene when expressed in an engineered *E. coli* strain, contrasting with the accumulation of lycopene as the most desaturated molecule found in the natural host. Such a discrepancy led us to hypothesise that CrtI enzyme activities may depend on the experimental conditions and thus be inconsistent with the patterns generated in the natural host. Unfortunately, assays of phytoene desaturases activities after heterologous expression were performed in different assay conditions which impedes their direct comparison across various publications^16,21,23^.

Here, we present a comparison of the natural evolution and kinetic properties of selected CrtI enzymes expressed and assayed under standardised conditions. Our results show that potentially all CrtI enzymes can catalyse desaturation reactions that progress beyond the already observed end-products and that the pattern of products formed originates from variations in the reaction rates rather than affinity constants. We also show that, in vivo, the presence of lycopene cyclase modifies the carotene patterns generated by CrtI enzymes alone, partially mimicking the situation observed in a natural host.

## Results

### Relationship between genetic and functional evolution of certain CrtI enzymes

Sequence diversification between organisms may be accompanied by changes in enzyme activity and/or specificity. We thus compared the evolution of phytoene desaturases with their reported patterns of desaturated products found in natural hosts. However, not all phytoene desaturases perform the exact same chemical reaction. Notably, the plant PDS (EC 1.3.99.29) and cyanobacteria CrtP (EC 1.3.5.5) enzymes can only perform desaturation reactions up to di-*cis*-*ζ*-carotene, they do not have isomerase activity, and their evolution is divergent (Supplementary Fig. S1)^24^. Therefore, we focused our analysis on phytoene desatureses of the CrtI family. From among the 190 CrtI sequences (EC 1.3.99.29, 1.3.99.28, 1.3.99.31 and 1.3.99.30) described in Klassen^25^, we selected 28 representatives of different phylogenetic clades possessing them (20 from bacteria and 8 from fungi) for which carotene profiles produced in the natural host or in recombinant strains are known (Supplementary Table S2). To simplify the nomenclature of the enzymes studied, the phytoene desaturases are annotated hereafter as follows: a first letter for the name of the family (I for CrtI), followed by a digit that indicates the formally attributed maximal number of CrtI-induced double bonds and the first letters of the genus and species. I4_Pa thus corresponds to CrtI from *Pantoea ananatis*, which performs four desaturation reactions on phytoene in the natural host. The four numerals used also match the EC classification of CrtI enzymes as EC 1.3.99.29, 1.3.99.28, 1.3.99.31, and 1.3.99.30, corresponding to phytoene desaturases that catalyse 2, 3, 4, and 5 desaturations, respectively.

**Figure 2:**
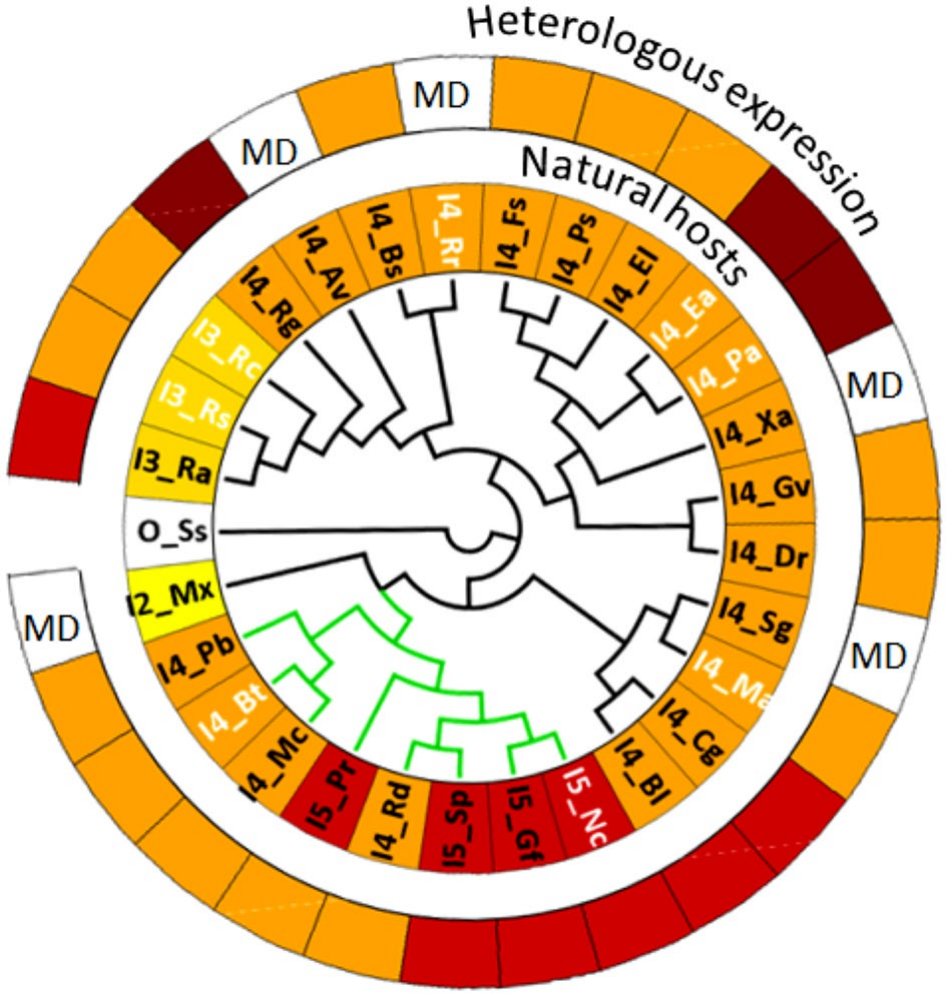
Phylogenetic tree of twenty-eight CrtI enzymes (Supplementary Table S2) from bacteria (black branch) and fungi (green branch). The inner and outer rings correspond to the maximal degree of unsaturation of the carotenes reported from natural hosts or heterologous expression data, respectively. Individual colours for each CrtI enzyme in the inner and outer rings correspond to the last desaturated product observed. Bootstrap values are presented in Supplementary Fig. S3. The phylogenetic tree was rooted using CrtO from *Synechocystis sp.* The eight names in white depict the enzymes studied in this work (I3_Rs, I3_Rc, I4_Rr, I4_Ea, I4_Pa, I4_Ma, I5_Nc, I4_Bt). The phylogenetic tree was generated using FigTree v1.4.3 (http://tree.bio.ed.ac.uk/software/figtree/). MD: missing data in the literature.

We performed an alignment of the 28 primary protein sequences and used it to build a phylogenetic tree using the maximum likelihood method (Fig. 2 and Supplementary Fig. S2). The reliability of the phylogenetic tree was validated by the strong boostrap value (100) of the clustering of eukaryotic phytoene desaturases. Two CrtI enzymes (I4_Gv and I2_Mx) were not clustered in their expected clade branch. This result is discussed in Supplementary Fig. S3. Overall, this tree shows similar clade organisation and congruence to that from the exhaustive phylogenetic study generated by Klassen^25^, although it was generated using only a subset of CrtI family members. We then examined whether the CrtI tree organisation could be linked with the respective enzyme function (inner ring in Fig. 2).

Certain bacteria produce carotenes containing only three double bonds (I3_Ra, I3_Rc, and I3_Rs), whereas only fungi produce molecules with up to five double bonds (I5_Pr, I5_Sp, I5_Gf, and I5_Nc). The individualization of the 3-step desaturases group does not correspond to the repartition of the *α*-proteobacteria phylum which appears to be clustered in 4 separate groups distributed randomly within the bacteria phylogenetic tree. While some sequence conservation between the eight CrtI of our study was visible (Supplementary Fig. S2), identification of specific sequence patterns associated with either one of the three families was not possible. However, when we used the 28 sequences, a clear individualization of the 3-step desaturases as a branch appeared even if it was not obvious when looking at the full sequence alignment. Thus, the separation of the 3-steps from the rest of the 4-steps enzymes seems concomitant with the apparition of a specific subgroup. It should be noted that 3- and 5-steps desaturases share at least 66% and 47% sequence identity, respectively. In contrast, the sequence identity of 4-steps desaturases can be as low as 27%. Furthermore, 4-steps desaturases are detected in different clades, independently of the organisms considered, suggesting that they may represent a common ancestor to all phytoene desaturases. We can thus hypothesise that 3- and 5-steps desaturases are apomorphic to lycopene-forming phytoene desaturases. Consequently, the ability to produce neurosporene or didehydrolycopene with 3- or 5-steps enzymes, respectively, may have arisen from the 4-steps ancestors. Overall, the assignation to any class does not simply depend on the global sequence identity to other classes but rather on specific motifs found in the primary sequence.

We then analysed the desaturation patterns of 23 of the 28 CrtI enzymes (outer ring in Fig. 2) determined after heterologous expression in bacteria. Eight of these phytoene desaturases can form carotenes containing a higher degree of unsaturation than those found in vivo, reaching lycopene for I3_Rs and I3_Rc, didehydrolycopene for I3_Ra, I4_Cg, and I4_Bl, and even tetradehydrolycopene for I3_Rg, I4_Ea, and I4_Pa. On the contrary, I5_Pr was found to produce only lycopene when expressed in *E. coli*, whereas torulene has been found in natural extracts of I5_Pr cultures^18,26^. Thus, there are discrepancies between the data obtained in vivo and that obtained after heterologous expression for approximately 25% of the analysed phytoene desaturases. We then decided to compare the activities of various CrtI enzymes (indicated in white in Fig. 2) from different clades either generated in vitro after expression in *E. coli* or in vivo in a synthetic carotene pathway reconstituted in yeast.

### Diversity of carotene patterns formed by several CrtI enzymes

**Figure 3:**
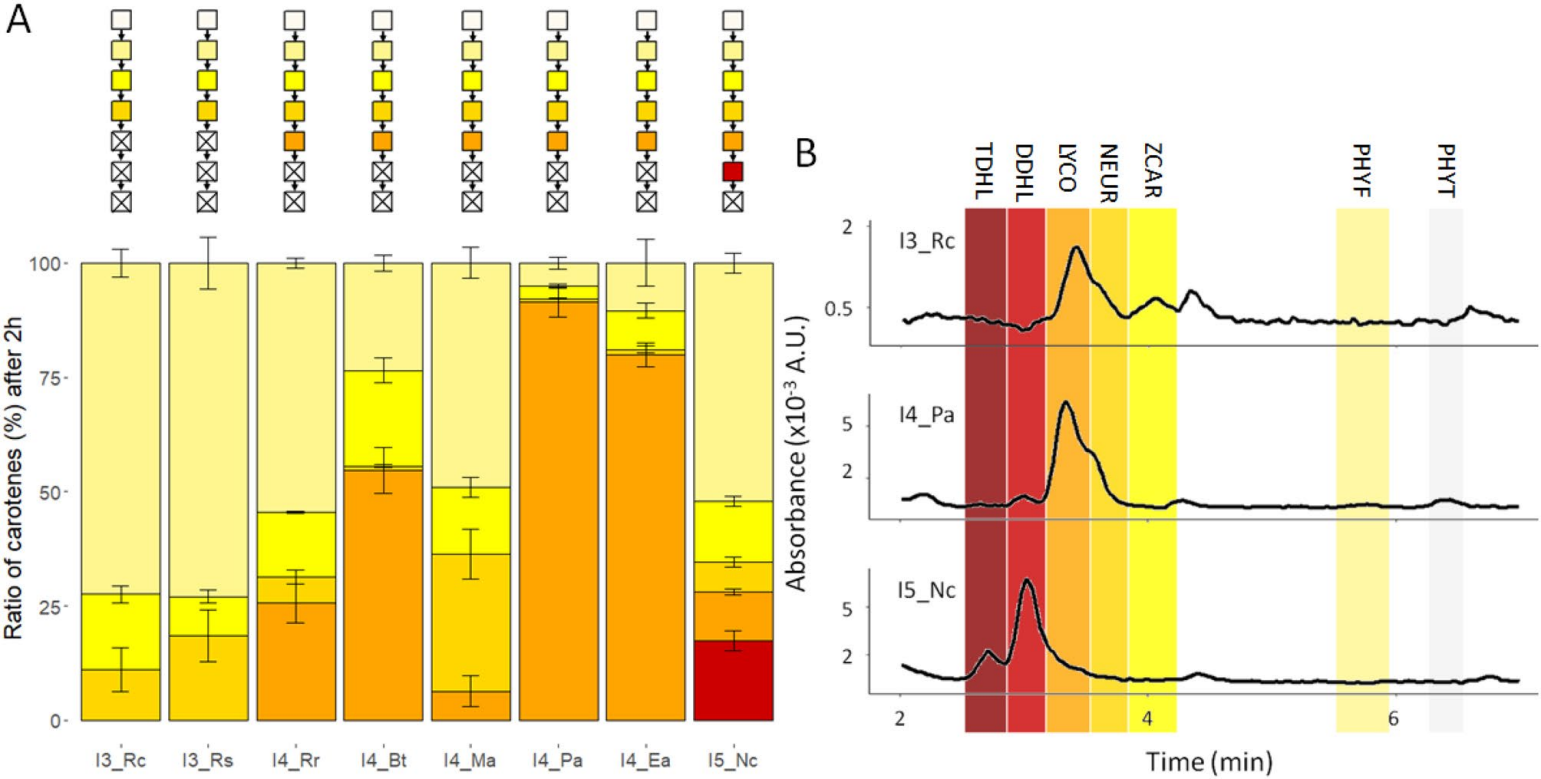
In vitro analysis of the carotenes produced in two or sixty hours by eight CrtI enzymes. Carotenes were analysed from enzymatic assays performed on the soluble fraction of crude extracts. (A) Top: schematic representation of the pathway, in which the coloured squares represent the various carotenes observed in the histograms (same colour coding as in Fig. 1), and the white crossed squares, carotenes that were not detected. Bottom: carotenes were quantified by HPLC–UV and are reported as the fraction of the total carotene content. (B) Sixty-hour assay HPLC–UV profiles at 495 nm (wavelength adapted for the detection of both didehydrolycopene and tetradehydrolycopene) of a subset of CrtI enzymes (I3_Rc, I4_Pa, and I5_Nc). Graphs were generated using RStudio v1.1.463 (https://rstudio.com/). TDHL, tetradehydrolycopene; DDHL, didehydrolycopene; LYCO, lycopene; NEUR, neurosporene; ZCAR, *ζ*-carotene; PHYF, phytofluene; PHYT, phytoene. Relative content are reported in Supplementary Table S3.

All open reading frames were cloned into the same expression vector (pUC_ara_) and expression assessed in the BL21-DE3 *E. coli* chassis strain. We developed a unique procedure, based on that described in Schaub et al*.*^19^, that allows measuring the desaturation activities of all tested CrtI enzymes in vitro. The soluble fraction of crude extracts of bacterial cultures expressing the CrtI enzymes was mixed with liposomes produced with soy lecithin containing phytoene and the FAD cofactor. The enzymatic reaction was then allowed to proceed for up to 60 h and the various authenticated carotenes quantified by HPLC–UV based on their retention times and specific spectra (Supplementary Fig. S4). There was a large disparity in the expression of the eight CrtI enzymes, leading to variations in the conversion of phytoene (Supplementary Fig. S5). Therefore, we only analysed the presence of the various desaturated phytoene products in the experiments performed on crude extracts rather than the specific conversion of each (Fig. 3).

As expected, all tested CrtI enzymes produced distinct patterns of unsaturated molecules, with the most highly unsaturated ones corresponding to those found in the natural hosts: neurosporene for I3_Rc and I3_Rs, didehydrolycopene for I5_Nc, and lycopene for the rest (inner ring of Fig. 2 and Fig. 3A). This may signify that the presence and distribution of desaturated phytoene products can be used for rapid and global characterisation of CrtI activities, even with variable amounts of the different enzymes observed under our experimental test conditions. Interestingly, the pattern of intermediate carotenes was quite different between the four-step CrtI enzymes. For example, the carotene pattern formed by I4_Pa and I4_Ea was similar, representing a group of CrtI enzymes that produce a high proportion of lycopene relative to the other intermediates (more than 75% of the desaturated phytoene products). I4_Rr and I4_Bt produced from 1:1 to 1:3 stoichiometries of lycopene to other desaturated phytoene products. On the contrary, I4_Ma formed relatively low amounts of lycopene (10% of the total desaturated products, Supplementary Fig. S5). Thus, although the assigned functional formal nomenclature of these lycopene-forming phytoene desaturases is coherent with their determined last product formed, the ratios between the various products may suggest a more complex classifications for CrtI (Fig. 2).

We next determined how the patterns and the last carotene formed change when increasing incubation (60 h, Fig. 3B and Supplementary Table S3). Lycopene was still the highest desaturated phytoene product detected for I4_Bt. As I4_Bt was the slowest phytoene-converting CrtI enzyme in our study (Supplementary Fig. S5), it is possible that the amount of lycopene produced is not sufficient to sustain a good conversion rate towards higher desaturated products. However, after 60 h, I3_Rc produced lycopene, I4_Pa didehydrolycopene and tetradehydrolycopene, confirming previously obtained results for these two enzymes when expressed in *E. coli* in the context of a reconstituted metabolic pathway^20^. I4_Ea and I5_Nc were able to perform six consecutive desaturation and thus produce tetradehydrolycopene, which is the first observation of the in vitro formation of such highly desaturated phytoene products by these two CrtI enzymes. We thus confirm that phytoene desaturases of the CrtI family can perform more desaturation reactions in vitro than those found in natural hosts.

### Kinetic analysis of product formation for a restricted set of CrtI enzymes

**Figure 4:**
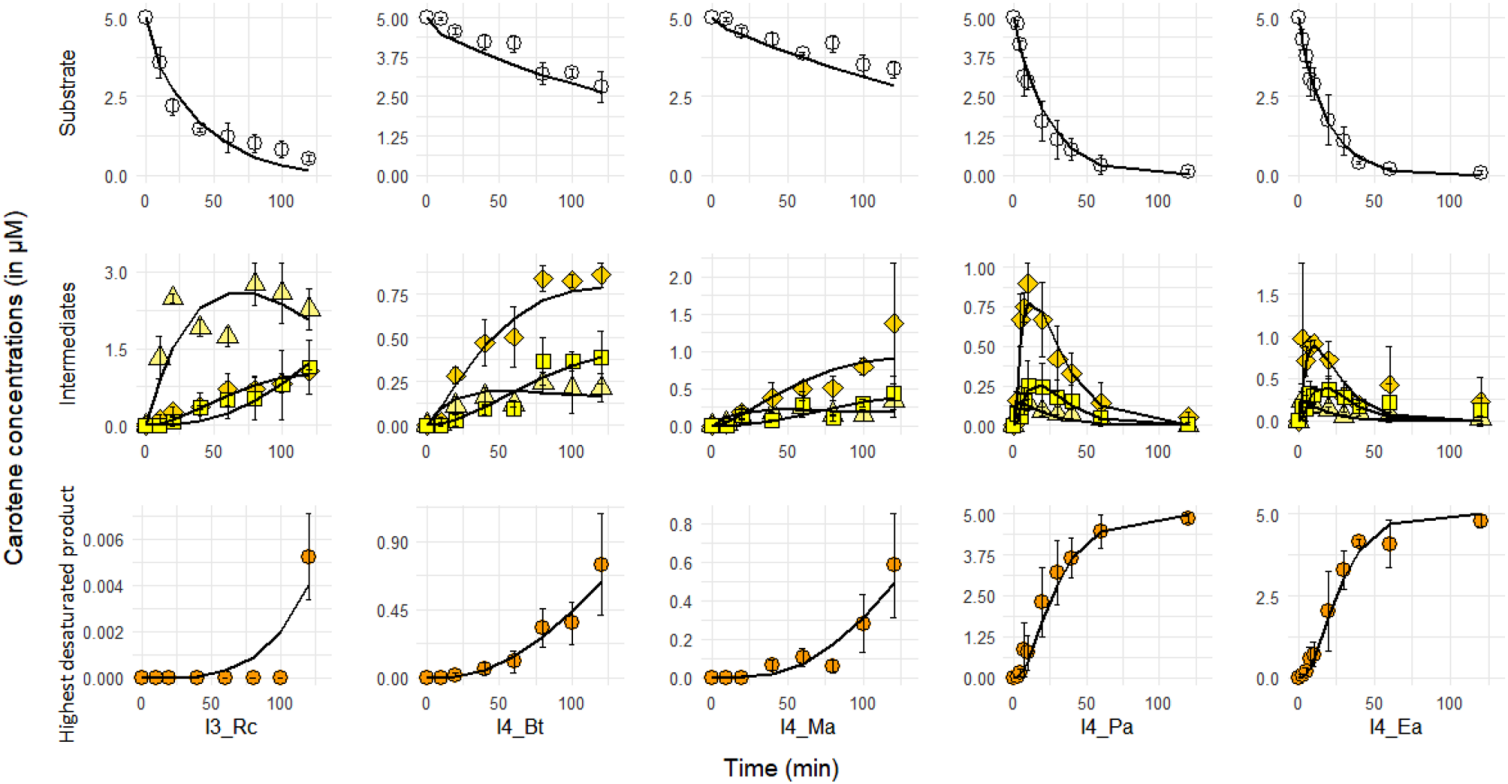
Analysis of the products formed during one hundred and twenty minutes with five purified CrtI enzymes expressed in *E. coli* (I3_Rc, I4_Bt, I4_Ma, I4_Pa, and I4_Ea). Carotenes were separated into three groups: substrate (phytoene), intermediates (phytofluene, *ζ*-carotene and neurosporene) and highest desaturated product (lycopene). Data points are shown as symbols coloured according to the previously used colour scheme: phytoene, white circles; phytofluene, triangles; *ζ*-carotene, diamonds; neurosporene, squares; lycopene, orange circles. The solid black lines correspond to the fit obtained using COPASI software and an explicit Michaelis–Menten model. Schematic representation of reactions performed by phytoene desaturase is given in the Supplementary Equation (S1). Details of the differential equations are given in Supplementary Equation (S2). Dissociation constants and turnover values are given in the Supplementary Table S4. Graphs were generated using RStudio v1.1.463 (https://rstudio.com/).

We further analysed the desaturation reaction kinetic profiles of the same CrtI enzymes. Only five of the eight CrtI enzymes could be purified in an active form (I3_Rc, I4_Bt, I4_Ma, I4_Ea, and I4_Pa). We thus quantified the substrate disappearance (Fig. 4, top panels) and products formation (Fig. 4, middle and bottom panels) of these five purified CrtI enzymes. Although all tests were performed with the same quantity of enzyme, the rates of phytoene consumption fell into two categories: slow enzymes (I4_Bt and I4_Ma) and fast enzymes (I3_Rc, I4_Ea and I4_Pa). I4_Ea and I4_Pa gave the same pattern of intermediate formation over time, similar to that at two hours with crude extracts (Fig. 3) For these two enzymes, the quantity of phytofluene, *ζ*-carotene and neurosporene were maximal at 20 min, whereas lycopene reached its maximum after 60 min. For I3_Rc, the decrease in the phytoene concentration was similar to that found for I4_Pa and I4_Ea, but it was phytofluene that reached a maximum at 50 min rather than the final product (neurosporene), which continued to increase throughout the experiment. Lycopene was also formed by I3_Rc at the very end if the reaction, confirming our previous observation on crude extracts, although the very low amount probably precluded us from detecting it before (limit of detection of the HPLC method). I4_Bt and I4_Ma showed relatively similar kinetics for formation of the intermediates (phytofluene and neurosporene) and final product (lycopene), whereas the two enzymes were evolutionarily well separated in the phylogenetic tree.

We further analysed how these patterns may be linked to rates and/or affinity constants by building a simplified enzymatic model using COPASI software^27^. We hypothesised that each reaction can be fitted by a regular Michaelis–Menten equation with explicit binding of each product (phytofluene, *ζ*-carotene, neurosporene, and lycopene) to the enzyme and partitioning of the total enzyme concentration between the various enzyme–substrate/product complexes and free enzyme (please see schematic representation of reactions performed by phytoene desaturase used in COPASI, Supplementary Equation S1, and the referred differential equations generated by our model in COPASI, Supplementary Equation S2). The steps corresponding to the formation of didehydrolycopene and tetradehydrolycopene were not included in the model because they were not detected during the two-hour experiments.

Several independent fitting experiments were attempted, with or without constraints on the different rate constants (*k*_*f*_ and *k*_*r*_). The absence of constraints on these constants resulted in aberrant values. The application of constraints on the three rate constants (maximal or minimal values) resulted in better quality fitting until the range explored was too stringent and the fitting was again of poor quality. Values for *k*_*f*_ were between 1 and 0.1 µM^−1^.s^−1^ and those for *k*_*r*_ between 1 and 10 s^−1^, leading to *K*_*d*_ values ranging from 1 to 100 µM, representing the best compromise we could manage. There is no report in the literature of any of these *K*_*d*_ values, which could serve as a basis for comparison and hence the values we obtained should be interpreted with caution, although the range explored can be considered to be reasonable. The obtained fitting were of generally good quality (Fig. 4), except for lycopene production by I3_Rc, which is again likely due to the limit of detection for lycopene. *K*_*d*_ and *k*_*cat*_ values for each of the reaction are presented in the Supplementary Table S4. Differences in the *K*_*d*_ and *k*_*cat*_ values followed the grouping determined empirically with the time course of product formation. For example, I4_Ea and I4_Pa have approximately the same *K*_*d*_ and *k*_*cat*_ for each of the reactions, as did I4_Ma and I4_Bt. However, the *k*_*cat*_ of the reaction leading to *ζ*-carotene was one order of magnitude greater for I4_Bt than I4_Ma, reflecting the faster production of the latter intermediate with CrtI from *Blakeslea trispora*. Overall, the variation in the dissociation constants, as well as turn-over, was relatively high between the CrtI enzymes (100-fold range for *K*_*d*_ and 300-fold range for *k*_*cat*_). This is the first report of *K*_*d*_ and *k*_*cat*_ values for the various reactions catalysed by phytoene desaturases calculated directly from kinetic experiments without using multiple intermediate substrates. Although the Michaelis–Menten model we used does not take into account possible features of plant phytoene desaturases, such as possible multimeric forms accompanied by substrate exchange between monomers during successive reactions^10^, we believe our model shows that a relatively simple mechanism can explain how the various patterns of carotenes generated by CrtI can arise from relatively large variations in *K*_*d*_, *k*_*cat*_ or both.

### CrtI-generated carotene patterns are modified in vivo in a full *β*-carotene pathway

**Figure 5:**
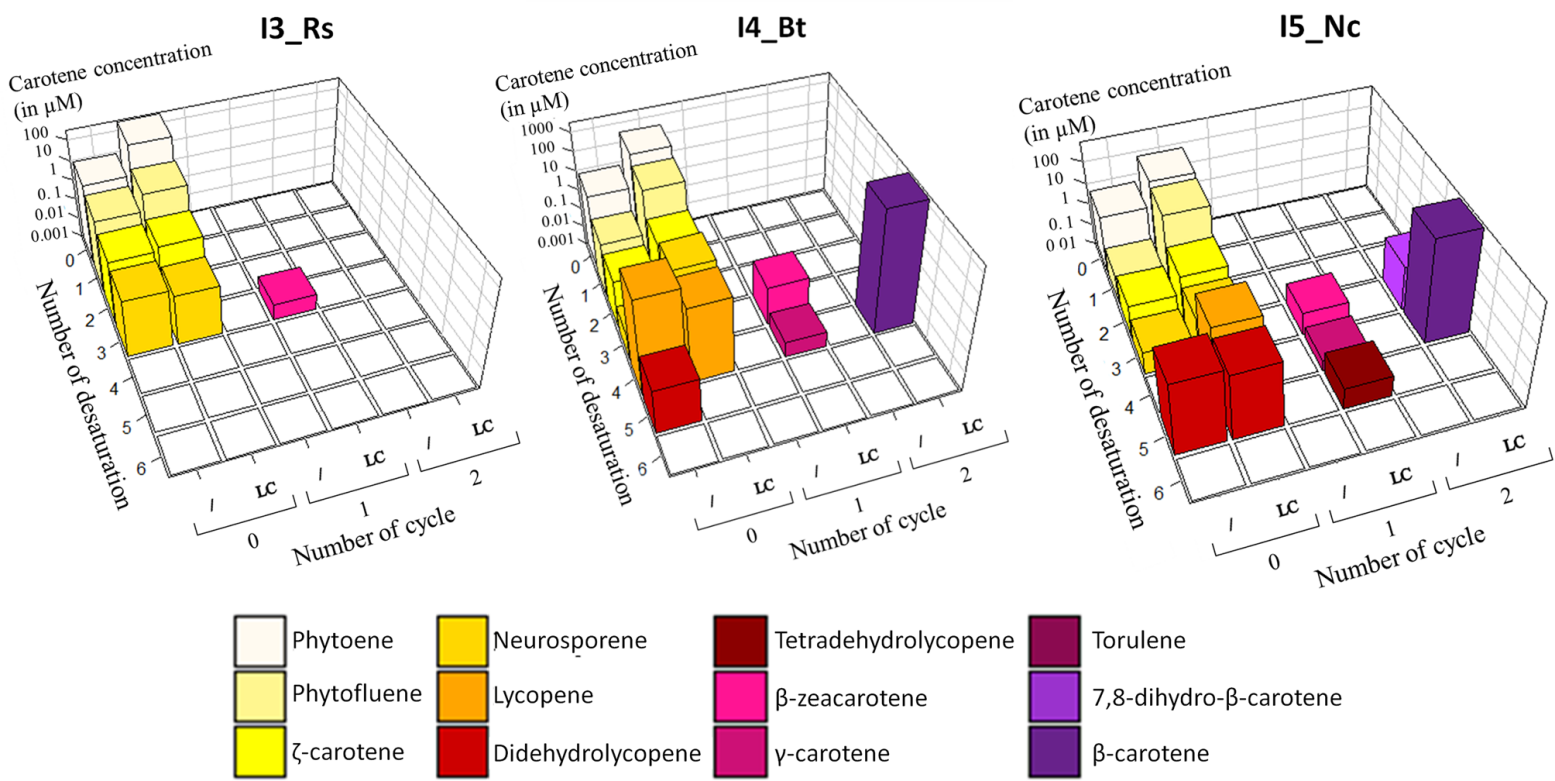
Patterns of carotenes generated in vivo for three phytoene desaturases (I3_Rs, I4_Bt and I5_Nc) associated with phytoene synthase only (/) or phytoene synthase and lycopene cyclase (LC). The X and Y axes represent the number of desaturations and cycles, respectively. The Z axis (logarithmic scale) corresponds to the mean concentrations (three independent experiments). Each carotene is thus represented by a single bar with the previously used colour coding. Values and standard deviations are also reported in the Supplementary Table S5. Graphs were generated using RStudio v1.1.463 (https://rstudio.com/).

Finally, we expressed a representative of each of the major CrtI groups (I3_Rs, I4_Bt, and I5_Nc) in a completely different context to further understand their influence on the carotene pathway, especially that of lycopene cyclase. We used two *S. cerevisiae* strains expressing either the bifunctional phytoene synthase/lycopene cyclase CrtYB from *P. rhodozyma* (YHR002) or only its truncated phytoene synthase domain (tCrtB, YHR001)^28^. Carotene quantification was performed after 48 h of culture under conditions in which accumulation of the different carotenes was maximum in our strains^28^, mimicking the conditions regularly used for the in vivo determination of carotene production in natural hosts (Fig. 5 and Supplementary Table S5)^21,29,30^.

We first analysed carotene production in the absence of the lycopene cyclase (Fig. 5). Remarkably, for I3_Rs, distribution of the various carotenes was similar to that observed using crude extracts containing I3_Rs and in vitro activity measured after two hours: phytofluene was the major intermediate formed (approximately 70%), while *ζ*-carotene and neurosporene represented approximately 20% and 10%, respectively (Fig. 5 compared to Fig. 3). On the contrary, for I4_Bt and I5_Nc, the stoichiometries between the carotenes were very different from those found in vitro after two hours of incubation, with both enzymes producing much more lycopene and didehydrolycopene than the other intermediates. The difference of behavior between I3_Rs, on one hand, and I4_Bt and I5_Nc, on the other, cannot be attributed to the phytoene content, which was similar for the three strains. It thus probably arises from differences in the heterologous expression (cell type, expression, presence of cellular compartment, etc.). In the case of I4_Bt, didehydrolycopene could be detected in the cellular fraction, which again shows that CrtI enzymes can form highly unsaturated molecules, depending on the culture/conversion conditions.

In the presence of CrtI enzymes, phytoene synthase, and lycopene cyclase (full length CrtYB), there was a general increase in the total carotene concentration (Fig. 5). This was also true for phytoene, again confirming the positive role of the lycopene cyclase domain of CrtYB in increasing the general metabolic flux toward carotenes^28^. This general trend was also accompanied by major changes in carotenes repartitioning. For example, the quantity of neurosporene was slightly lower and *β*-zeacarotene was produced in the strain containing I3_Rs and CrtYB. The same was true for the strain containing I5_Nc and CrtYB, in which the amount of the last desaturated product (didehydrolycopene) decreased while, in parallel, torulene was produced. Furthermore, the presence of lycopene cyclase with I5_Nc allowed the visible accumulation of lycopene. The situation was more complex for the strain containing I4_Bt and CrtYB, as didehydrolycopene completely disappeared but there were no concomitant production of torulene, suggesting strong rerouting of the carotene pathway.

There was a good correlation between the number of desaturation introduced by the CrtI enzymes and one-cycle carotenes (*β*-zeacarotene for I3_Rc, *γ*-carotene for I4_Bt, and torulene for I5_Nc) but not two-cycle carotenes (dihydro-*β*-carotene for I3_Rc and *β*-carotene for I4_Bt). Overall, the data obtained in vivo clearly demonstrate that lycopene cyclase greatly perturbs the pattern of product formation by competing at different steps of the desaturation reactions. The push–pull effect of lycopene cyclase can completely remodel the pathway, enabling the accumulation of certain molecules while making others disappear. This behaviour is dependent on the CrtI enzyme, as well as the concentration of the carotene metabolites.

## Discussion

Phytoene desaturases of the CrtI family encompass four enzymatic functional subgroups (EC 1.3.99.29, EC 1.3.99.28, EC 1.3.99.31 and EC 1.3.99.30), which correspond to *ζ*-carotene, neurosporene, lycopene, and didehydrolycopene-forming enzymes, respectively^15^. Here, we present an analysis of eight members of CrtI family, starting from the phylogenetic clustering of 28 enzymes from the four groups, analysing the in vitro-generated products for some, and finally assessing the role of lycopene cyclase in modifying phytoene synthases-generated carotene patterns using an in vivo metabolic engineering approach. Given that 4-steps enzymes are by far the most abundant and probably evolutionary preceded 3- and 5-steps enzymes, it is reasonable to trace the evolutionary path of the diversification of CrtI enzymes using 4-step enzymes. However, 4-step enzymes do not constitute a homogeneous group and our in vitro data, generated using crude extracts or purified enzymes, clearly demonstrate the existence of subgroups based on the generated carotene profiles. For example, I4_Ea and I4_Pa or I4_Bt and I4_Ma form two distinct subgroups that possess the same kinetic characteristics, as determined by analysing their carotene product profiles over time. Although the grouping between I4_Ea and I4_Pa is quite evident based on the phylogenetic tree, I4_Bt and I4_Ma are phylogenetically quite distant.

Functional characterisation of CrtI enzymes after heterologous expression was achieved in many studies^17,19,20,31–33^. Their capacity to perform more degrees of unsaturation than their attributed activities was also demonstrated for I4_Pa and I5_Nc^19,20,33,34^. Our study generalise the fact that promiscuity in CrtI enzymes is actually a quite distributed property: 3-, 4- and 5-steps enzymes may all be capable of performing desaturations up to tetradehydrolycopene. The real patterns of carotene produced will then only depend on other independent factors that we point out in our study: the biological conditions in which they are assayed (in vitro after heterologous expression, in vivo in reconstituted synthetic pathways and in vivo in the natural hosts), the modulation of the rates of transformation for each substrate and the presence of competing enzymes in the system such as lycopene cyclase but also phytoene synthase as demonstrated by others^20^.

The determination of rate and binding constants for the different reactions was performed using kinetic analysis and an Ordinary Differential Equations modelling software (COPASI^27^). This type of analysis is extremely powerful and was used successfully to explore how association between monomers of rice PDS was coherent with the kinetic profiles of the products formed^10^. Here, using a simple Michaelis–Menten model, we were able to derive a series of *K*_*d*_ and *k*_*cat*_ values for each of the reaction up to lycopene of the studied CrtI enzymes. While these values cover a large range of dissociation and rate constants, they are within the ranges already obtained for CrtI enzymes^10,17,19,35^. The values obtained can probably be further refined with the study of individual reactions with each of the various substrates/products. However, owing to the relative simplicity of the setup, the methodology used herein is adequate for rapidly screening assay conditions and major differences between various CrtI isoforms.

Differences of carotene patterns due to variable in vitro assay conditions are easily understandable. Variation of expression levels and loss of activity upon purification are known factors influencing desaturase activities^36^. Hydrophobicity of the substrate and products as well as the association between enzyme and membrane can modulate the optimal environment of the enzymatic reaction^17,19,37,38^. CrtI from *Pantoea ananatis* associates spontaneously to liposomal membranes^19^ but no membrane-spanning region per se was evidenced, suggesting a monotopic binding to membranes. Unfortunately, little is known about specific lipid requirements of CrtI enzymes, although the mode of membrane association would argue against lipid specificity. Natural carotenogenic enzyme fusion exists in nature between phytoene synthase and lycopene cyclases^18^ or between all three enzymes^39^, potentially leading to higher efficiencies of the whole pathway. New-to-nature fusions between the three enzymes also demonstrated higher efficiencies than natural proteins but also variations in the carotenoid patterns produced^28^. Therefore, membrane association of CrtI enzymes may also be in part promoted by association with its partners, in particular CrtY which is an integral membrane protein. Our in vitro enzymatic assays were performed on liposomes and without other proteins which may change the association of CrtI to liposomes. In higher plants, PDS enzymes (converting *cis*-phytoene to tris-*cis*-*ζ*-carotene) form homotetramers and these superstructures are important for the PDS function^10,40,41^. On the contrary, multimers of CrtI enzymes were never detected and the sole X-ray structure of CrtI describes a perfectly monomeric form. However, it was suggested that CrtB, CrtI, and CrtY enzymes could form a protein complex at the membrane^18,37^. In the proposed carotenogenic complex, two transmembrane lycopene cyclases assemble with two phytoene synthases and then recruit four phytoene desaturases.

The potential oligomeric formation of CrtI complex may also be affected by the presence of the polar polyhistidine-tag in the C-terminus of the protein. Due to its relatively small size, this amino acid motif is not believed to significantly interfere with the function and structure of most proteins. However, the tag may affect the oligomeric states of proteins^42,43^ and the production of membrane proteins^44^. Nonetheless, PDS homotetramers were crystallized using protein tagged with polyhistidine tags and the membrane association of such complex was observed^41^, arguing against a strong unfavourable role of it for the association between CrtI and the liposomes.

Finally, we also confirm that carotene patterns are highly dependent, in vivo, on the interactions of CrtI with lycopene cyclase. In vivo, the close proximity between the cyclase and the desaturase may promote a very efficient transfer of various intermediates to lycopene cyclase^28,33^. Here, we show that lycopene cyclase can also modify the equilibrium between the various desaturation reactions. For example, didehydrolycopene is formed by I4_Bt in the absence of lycopene cyclase. However, this highly desaturated intermediate as well as its potential cyclized derivative (torulene) are completely absent in the presence of CrtYB and the flux is completely rerouted toward the production of *β*-carotene. Furthermore, the introduction of cycles at one extremity may ultimately prevent further desaturation^37,45^. Various studies have proposed a supra molecular assembly of the carotenogenic enzymes^18,37^ that could enhance the efficiency of the pathway by a strong push in the total flux. We now hypothesise that the potential presence of these assemblies or, on the contrary, their absence in the case of heterologous expression, also change the pattern of carotenes made by either enhancing or inhibiting particular desaturation reactions. As such, the carotene pattern that can be seen in vivo in natural hosts is a rather imperfect representation of the real capacity of phytoene desaturases to produce certain intermediates. Our work enlightens that the actual enzymatic nomenclature of CrtI is probably not a strong indication of the real activity of phytoene desaturases. We hypothesise that the sub-grouping into the four EC categories might not be adapted, at least not based on the theoretical last product formed in vivo, in natural hosts in which the presence of the full *β*-carotene pathway complicates the true CrtI function’s attribution.

Biotechnological approach for the production of any of the potentially interesting intermediate molecule is of crucial importance, not only because of the potential scarcity of natural sources, but also because of the lack of potential tools to rewire the stoichiometries between the various carotenes in the pathway^46^. Heterologous expression in various microbial hosts is evidently an asset to alleviate these known drawbacks. The large range of possible combinations of phytoene synthase, phytoene desaturases and lycopene cyclases from diverse origins associated with different heterologous systems for in vitro or in vivo production can certainly expand the portfolio of possible carotenes for health, food or feed applications^47^. Overall, even if phytoene desaturases may represent a quite original enzymatic case, our results demonstrate the need for thorough biochemical analyses of enzyme activities, in standardised heterologous expression and in vitro activities conditions, to fully attribute enzyme functions (or nomenclature). Clearly, phylogenetic data associated with metabolites profiles are not sufficient to reflect the amazingly large diversity of enzyme functions in large families.

## Methods

### Phylogenetic methods

All amino acid sequences were aligned using the MUSCLE algorithm of the SeaView software v4.5.4 (http://doua.prabi.fr/software/seaview). Phylogenetic analyses were conducted primarily using the Tree Inference features of the IQ-TREE software v1.6.11 as implemented through the CIBIV web portal (http://iqtree.cibiv.univie.ac.at/). In all cases the Le-Gascuel (LG) substitution matrix was used with maximum likelihood approach, amino acid frequencies were determined empirically from the data, the proportion of invariant sites was estimated automatically, the site rate variation was following a gamma distribution with four rate categories. Best scoring tree is used for visualisation and bootstrap values are calculated for each node. Preliminary Tree Inference experiments using other substitution matrices (WAG, BLOSUM62, JTT) gave equivalent results. Further experiments using parsimony (PROTPARS) and distance (BioNJ, Poisson) tree construction methods implemented in SeaView also yielded congruent results. Trees were rooted using *β*-carotene ketolase (CrtO) from *Synechocystis sp* (A0A068N4T0, annotated O_Ss) as an outgroup enzyme.

### Bacterial plasmids and *E. coli* strains

For the heterologous expression of phytoene desaturases in *E. coli*, the different genes were codon optimized prior synthesis (removing unusual codons), associated with a hexahistidine-tag at their C-termini and cloned in pUC_ara_ vector^48^. Protein sequences were directly assessed from the Uniprot numbers (https://www.uniprot.org/) described in the text. DNA sequences were back translated from the protein sequences. Codon optimization was performed using the codon optimized tool from the IDT web site (https://www.idtdna.com). The optimized DNA sequences are available in the Supplementary Table S6. We used commercial DH5*α* and BL21(DE3) from NEB, respectively for cloning steps or protein expression steps. We used the commercial transformation protocol of this company.

### *Escherichia coli* media and bacterial culture

LB plus ampicillin (100 µg/mL) was used for cloning, transformation, plasmid purification and pre-culture steps in bacteria. LB is composed by 1% of tryptone, 0.5% of yeast extract and 0.5% of NaCl. For heterologous expression of phytoene desaturases in bacteria, we used the medium ZYM5052^49^ supplemented with 100 µg/mL ampicillin (Sigma), 0.05% arabinose (Formedium) and 5 µM riboflavin (Sigma). Cells were grown at 37 °C until OD of 0.6, then the culture was switched to 20 °C overnight to allow for protein expression. ZYM5052 is composed by 1% tryptone (Formedium), 0.5% yeast extract (Formedium), 25 mM Na_2_HPO_4_ (VWR), 25 mM KH_2_PO_4_ (Fluka), 50 mM NH_4_Cl (Normapur), 5 mM Na_2_SO_4_ (SDS), 0.5% glycerol, 0.05% glucose, 0.2% *α*-lactose (Sigma), 2 mM MgSO_4_ (Prolabo), 50 µM FeCl_3_ (Sigma), 20 µM CaCl_2_ (Sigma), 10 µM MnCl_2_ (Sigma), 10 µM ZnSO_4_ (Sigma), 2 µM CoCl_2_ (Sigma), 2 µM CuCl_2_ (Sigma), 2 µM NiCl_2_ (Sigma), 2 µM Na_2_MoO_4_ (Sigma), 2 µM Na_2_SeO_3_ (Sigma) and 2 µM H_3_BO_3_ (Sigma).

### Phytoene and didehydrolycopene extraction and purifications

Phytoene from Carotenature was used to make a standard curve with HPLC–UV, while purified phytoene (done in this study) was used for enzymatic reactions. Phytoene was extracted using acetone from *S. cerevisiae* strain, YHR02, after three-days culture in YPG at 30 °C and 220 rpm. Solvent fraction was dried and resuspended in hexane, while concentration was determined from absorbance at 287 nm. Didehydrolycopene was purified from extraction of I5_Nc enzymatic reaction after sixty hours using preparative HPLC–UV. The concentration was determined from absorbance at 490 nm.

### Substrate preparation

2.5 mg of L-*α*-phosphatidylcholine from soybean (Sigma) were suspended in 0.5 mL of chloroform (Normapur) containing 50 nmoles of phytoene (purified in our study), slightly dried under a nitrogen stream and suspended in buffer I (50 mM of Tris(hydroxymethyl)aminomethane at pH 8.0, 100 mM of KCl, 1 mM of Tris(2-carboxyethyl)phosphine) in the presence of 100 *µ*M FAD. The solution was sonicated during 1 min at 60% on ice to form small vesicles of liposomes.

### In vitro assays

Experiments using crude extracts were carried out as follows. First, 10 mL cultures of *E. coli* expressing the desaturases were centrifuged at 8,000 × *g*, the cell pellets suspended in 900 *µ*L buffer I supplemented with 1 mM TCEP, and 100 *µ*L substrate added (*i.e.* 5 nmol of phytoene). The solution was sonicated three times at 30% for 45 s on ice. Enzymatic reactions were carried out at 30 °C with 800 rpm agitation for 2 or 60 h in the dark and stopped by adding one volume of chloroform/methanol (2:1, v/v) and immediately vortexing for 30 s. After centrifugation at 20,000 × *g* for 5 min, the organic phases were dried under a nitrogen flux and suspended in 50 *µ*L hexane (VWR) before HPLC–UV analysis.

### Analytical method

An HPLC module (Waters 2690) combined to a UV detector (Waters 996) and a PerkinElmer column (0711–0015, RP18, 100 × 4.6 mm) coupled to a pre-column (0711–0092, 15 × 3.2 mm) was used. Gradient started with 30% of TBME (*tert*-butyl methyl ether, Sigma) and 70% of acetonitrile/water (90:10, Scharlau) to finish at 10 min with 35% of TBME, then 100% of TBME during 2 min and initial conditions for 3 min. Flow was maintained at 1 mL/min, the temperature of the column was at 30 °C during runs and samples were stored at 10 °C prior injection. Quantification of carotenes was based on their reported molar extinction coefficients (Supplementary Table S1) except for tetradehydrolycopene and dehydro-*β*-carotene for which the molar extinction coefficients of the closest molecules (didehydrolycopene and *β*-zeacarotene, respectively) were used to estimate their quantities.

### Protein purification

*Escherichia coli* cells lysis was performed at 4 °C after resuspension of cell pellets from 500 mL culture in buffer I and using a French press (3 passages). After centrifugation at 10,000 g during 15 min, the supernatant was applied onto a TALON superflow resin (Takara). Buffer I was further applied until absorbance at 280 nm reached 0.1. Washing steps were performed with buffer I containing 10 mM imidazole (Euromedex). Proteins were eluted with buffer I containing 150 mM imidazole and 100 µM FAD (Sigma). Eluted fractions containing phytoene desaturases were concentrated with a Merck centrifuge unit (MWCO 30 kDa) at 4,000 g during 15 min at 4 °C, followed by dialysis (1:500, v:v) against buffer I containing 100 µM FAD during 30 min under agitation at 4 °C (Pur-a-lyzed dialysis kit, MWCO 3,5 kDa, Sigma). A second concentration step was performed using an Amicon centrifuge unit (MWCO 30 kDa) at 4 °C until 500 *µ*L reached. Protein content was analyzed by SDS PAGE gel colored by InstantBlue stain (Expedeon) and the protein concentration was determined by the Pierce method (Thermoscientific). Purified proteins were aliquoted by amounts of 25 µg and stored at −80 °C until use.

### Kinetics of purified enzymes

25 µg of proteins, 400 *µ*L of buffer I containing 100 µM FAD and 100 *µ*L of substrate were incubated as previously described. Enzymatic reactions were stopped as described after 0, 2.5, 5, 7.5, 10, 20, 30, 40, 60 and 120 min for I4_Pa and I4_Ea or at 0, 10, 20, 40, 60, 80, 100 and 120 min for the rest of the phytoene desaturases.

### Modelling of kinetics data

The biochemical model corresponding to Supplementary Equation (S1) was implemented in COPASI software v4.29 (http://copasi.org/). The parameter estimation of *k*_*f*_, *k*_*r*_ and *k*_*cat*_ constant rates was using experimental date obtain during the kinetic assay of purified enzymes. The mean square method was used to normalise weights per experiment. We set lower and upper value for each constant rate as follow: *k*_*f*_, from 0.1 to 1 µM^−1^.s^−1^, *k*_*r*_, from 1 to 10 s^−1^ and *k*_*cat*_, from 10^–5^ to 0.1 s^−1^. The particle swarm with 2000 iterations was used.

### Fungal plasmids and *S. cerevisiae* strains

The exact same coding sequences were used for cloning into pYeDP60^50^ vector for *S. cerevisiae* expression, in association with a FLAG-tag instead of the previous hexahistidine-tag. We used YHR01 and YHR02 from Rabeharindranto et al*.*^28^, using the protocol for yeast transformation adapted from Gietz et al*.*^51^. YHR01 and YHR02 correspond to *S. cerevisiae* BY4741 engineered for GGPP production (over-expression of GGPP synthase, CrtE, from *P. rhodozyma* and truncated form of HMG-CoA reductase, HMG1)^28^. Phytoene production in YHR02 is mediated by the expression of bifunctional phytoene synthase/lycopene cyclase, CrtYB and in YHR01 thanks to the expression of the truncated form of CrtYB having only the phytoene synthase activity. Thus, the lycopene cyclase activity is only present in YHR02 strain. For yeast growth, minimal media (without uracil) was used during the transformation and preculture processes. For carotenoid production, the cells were grown in YPG during 3 days at 30 °C.

### *Saccharomyces cerevisiae* media and fungal culture

SD-Ura is composed by 0.67% YNB with ammonium sulfate (Formedium), 2% of glucose and 0.077% of CSM-Ura (MP Bio). YPD and YPG are composed by 2% of peptone, 1% yeast extract and 2% of, respectively, glucose (Formedium) or galactose (Formedium). For in vivo assay, 3 days of culture at 30 °C and 220 rpm was used in YPG.

### In vivo assays

After expression, *S. cerevisiae* cells were centrifuged and resuspended in an equivalent volume of glass beads (0.5 mm in diameter, Sigma) and 2 mL acetone (VWR). The suspension was homogenised with a FastPrep apparatus (ThermoSavant) using three 45 s cycles, with the agitation set to 5.5. After centrifugation at 20,000 × *g* for 5 min, the solvent fraction was collected and a second and third solvent extraction performed. The combined solvent fractions were dried under a nitrogen stream and suspended in 100 *µ*L hexane.

## Supplementary information

Supplementary Information 1.

## References

[1] Britton G, Young AJ, Britton G (1993). Structure and nomenclature of carotenoids. Carotenoids in Photosynthesis.

[2] Krinsky NI (1998). The antioxidant and biological properties of the carotenoids. Ann. N. Y. Acad. Sci..

[3] Stange C (2016). Carotenoids in Nature: Biosynthesis, Regulation and Function.

[4] Frank HA, Polívka T, Hunter CN, Daldal F, Thurnauer MC, Beatty JT (2009). Energytransferfromcarotenoidstobacteriochlorophylls. ThePurplePhototrophicBacteria.

[5] Galasso C, Corinaldesi C, Sansone C (2017). Carotenoids from marine organisms: biological functions and industrial applications. Antioxidants.

[6] Kot AM, Błażejak S, Gientka I, Kieliszek M, Bryś J (2018). Toruleneandtorularhodin:“new”fungalcarotenoidsfor industry?. Microb. Cell Fact..

[7] Rodriguez-Concepcion M (2018). A global perspective on carotenoids: Metabolism, biotechnology, and benefits for nutrition and health. Prog. Lipid Res..

[8] Armstrong GA (1994). Eubacteria show their true colors: genetics of carotenoid pigment biosynthesis from microbes to plants. J. Bacteriol..

[9] Liang M-H, Zhu J, Jiang J-G (2018). Carotenoids biosynthesis and cleavage related genes from bacteria to plants. Crit. Rev. Food Sci. Nutr..

[10] Koschmieder J (2017). Plant-type phytoene desaturase: functional evaluation of structural implications. PLoS ONE.

[11] Sieiro C, Poza M, De Miguel T, Villa T (2003). Genetic basis of microbial carotenogenesis. Int. Microbiol..

[12] Maresca JA, Romberger SP, Bryant DA (2008). Isorenieratene biosynthesis in green sulfur bacteria requires the cooperative actions of two carotenoid cyclases. J. Bacteriol..

[13] Steiger S, Jackisch Y, Sandmann G (2005). Carotenoid biosynthesis in *Gloeobacter violaceus* PCC4721 involves a single crtI-type phytoene desaturase instead of typical cyanobacterial enzymes. Arch. Microbiol..

[14] Tsuchiya T (2005). The cyanobacterium *Gloeobacter violaceus* PCC 7421 uses bacterial-type phytoene desaturase in carotenoid biosynthesis. Febs Lett..

[15] Sandmann G (2009). Evolution of carotene desaturation: the complication of a simple pathway. Arch. Biochem. Biophys..

[16] Steiger S, Astier C, Sandmann G (2000). Substrate specificity of the expressed carotenoid 3, 4-desaturase from *Rubrivivax gelatinosus* reveals the detailed reaction sequence to spheroidene and spirilloxanthin. Biochem. J..

[17] Raisig A, Bartley G, Scolnik P, Sandmann G (1996). Purification in an active state and properties of the 3-step phytoene desaturase from *Rhodobacter capsulatus* overexpressed in *Escherichia coli*. The J. Biochem..

[18] Verdoes JC (2003). Metabolic engineering of the carotenoid biosynthetic pathway in the yeast *Xanthophyllomyces dendrorhous* (*Phaffia rhodozyma*). Appl. Environ. Microbiol..

[19] Schaub P (2012). On the structure and function of the phytoene desaturase CrtI from *Pantoea ananatis*, a membrane- peripheral and FAD-dependent oxidase/isomerase. PLoS ONE.

[20] Song GH, Kim SH, Choi BH, Han SJ, Lee PC (2013). Heterologous carotenoid-biosynthetic enzymes: functional complementation and effects on carotenoid profiles in *Escherichia coli*. Appl. Environ. Microbiol..

[21] Zhang J, Lu L, Yin L, Xie S, Xiao M (2012). Carotenogenesis gene cluster and phytoene desaturase catalyzing both three-and four-step desaturations from *Rhodobacter azotoformans*. FEMS Microbiol. Lett..

[22] Kim SH, Park YH, Schmidt-Dannert C, Lee PC (2010). Redesign, reconstruction, and directed extension of the *Brevibacterium linens* C40 carotenoid pathway in *Escherichia coli*. Appl. Environ. Microbiol..

[23] Stickforth P, Sandmann G (2007). Kinetic variations determine the product pattern of phytoene desaturase from *Rubrivivax gelatinosus*. Arch. Biochem. Biophys..

[24] Sandmann G (2002). Molecular evolution of carotenoid biosynthesis from bacteria to plants. Physiol. Plantarum.

[25] Klassen JL (2010). Phylogenetic and evolutionary patterns in microbial carotenoid biosynthesis are revealed by comparative genomics. PLoS ONE.

[26] Verdoes JC, Misawa N, van Ooyen AJ (1999). Cloning and characterization of the astaxanthin biosynthetic gene encoding phytoene desaturase of *Xanthophyllomyces dendrorhous*. Biotechnol. Bioeng..

[27] Hoops S (2006). COPASI—a complex pathway simulator. Bioinformatics.

[28] Rabeharindranto H (2019). Enzyme-fusion strategies for redirecting and improving carotenoid synthesis in *S. cerevisiae*. Metab. Eng. Commun..

[29] Hannibal L (2000). Isolation and characterization of canthaxanthin biosynthesis genes from the photosynthetic bacterium *Bradyrhizobium* sp .strain ors278. J. Bacteriol..

[30] Pasamontes L (1997). Isolation and characterization of the carotenoid biosynthesis genes of *Flavobacterium* sp. strain R1534. Gene.

[31] Xu Z, Tian B, Sun Z, Lin J, Hua Y (2007). Identification and functional analysis of a phytoene desaturase gene from the extremely radioresistant bacterium *Deinococcus radiodurans*. Microbiology.

[32] Dailey TA, Dailey HA (1998). Identification of an fad superfamily containing protoporphyrinogen oxidases, monoamine oxidases, and phytoene desaturase expression and characterization of phytoene desaturase of *Myxococcus xanthus*. J. Biol. Chem..

[33] Fraser PD (1992). Expression in *Escherichia coli*, purification, and reactivation of the recombinant *Erwinia uredovora* phytoene desaturase. J. Biol. Chem..

[34] Sandmann G, Woods WS, Tuveson RW (1990). Identification of carotenoids in *Erwinia herbicola* and in a transformed *Escherichia coli* strain. FEMS Microbiol. Lett..

[35] Albrecht M, Linden H, Sandmann G (1996). Biochemical characterization of purified *ζ*-carotene desaturase from *Anabaena* PCC 7120 after expression in *Escherichia coli*. Eur. J. Biochem..

[36] Chen F (2016). Small-molecule targeting of a diapophytoene desaturase inhibits *S. aureus* virulence. Nat. Chem. Biol..

[37] Hausmann A, Sandmann G (2000). A single five-step desaturase is involved in the carotenoid biosynthesis pathway to *β*-carotene and torulene in *Neurospora crassa*. Fungal Genet. Biol..

[38] Stickforth P, Sandmann G (2011). Structural and kinetics properties of a mutated phytoene desaturase from *Rubrivivax gelatinosus* with modified product specificity. Arch. Biochem. Biophys..

[39] Iwasaka H (2018). A possible trifunctional β-carotene synthase gene identified in the draft genome of *Aurantiochytrium* sp. strain kh105. Genes.

[40] Brausemann A (2017). Structure of phytoene desaturase provides insights into herbicide binding and reaction mechanisms involved in carotene desaturation. Structure.

[41] Gemmecker S (2015). Phytoene desaturase from *Oryza sativa*: oligomeric assembly, membrane association and preliminary 3d-analysis. PLoS ONE.

[42] Dudek HM (2010). Investigating the coenzyme specificity of phenylacetone monooxygenase from thermobifida fusca. Appl. Microbiol. Biotechnol..

[43] Abd Elhameed HA (2020). Modulation of the catalytic activity of a metallonuclease by tagging with oligohistidine. J. Inorg. Biochem..

[44] Cudowska B (2020). Production and use of recombinant profilins amb a 8, art v 4, bet v 2, and phl p 12 for allergenic sensitization studies. Molecules.

[45] Tian B (2008). Carotenoid 3, 4-desaturase is involved in carotenoid biosynthesis in the radioresistant bacterium *Deinococcus radiodurans*. Microbiology.

[46] Li C, Swofford CA, Sinskey AJ (2019). Modular engineering for microbial production of carotenoids. Metab. Eng. Commun..

[47] Sandmann G, Schrader J, Bohlmann J (2014). Carotenoids of biotechnological importance. Biotechnology of Isoprenoids.

[48] Furubayashi M, Li L, Katabami A, Saito K, Umeno D (2014). Construction of carotenoid biosynthetic pathways using squalene synthase. FEBS Lett..

[49] Studier FW (2005). Protein production by auto-induction in high-density shaking cultures. Protein Expr. Purif..

[50] Urban P, Truan G, Gautier J-C, Pompon D (1993). Xenobiotic metabolism in humanized yeast: engineered yeast cells producing human NADPH-cytochrome P-450 reductase, cytochrome b5, epoxide hydrolase and P-450s. Carcinogenesis (Lond.).

[51] Gietz D, St Jean A, Woods RA, Schiestl RH (1992). Improved method for high efficiency transformation of intact yeast cells. Nucl. Acids Res..

