# Supplementary material for: Multiplicity of carotene patterns derives from competition between phytoene desaturase diversification and biological environments
Source: Sci Rep. 2020 Dec 3;10:21106. doi: 10.1038/s41598-020-77876-4 (PMC7713294; doi:10.1038/s41598-020-77876-4)
Supplement: Supplementary file 1 — Supplementary Information 1. [file 41598_2020_77876_MOESM1_ESM.pdf]

Supplementary Information for
Multiplicity of carotene patterns derives from competition between phytoene desaturase diversification and biological environments

Mathieu Fournié[1,2,3] **and Gilles Truan**[1,*]

[1]TBI, Université de Toulouse, CNRS, INRA, INSA, Toulouse, France
[2]Adisseo France S.A.S., 10 place du Général de Gaulle, 92160 Anthony, France
[3]Groupe Avril, 11 Rue de Monceau, 75378, Paris, Cedex 08, France
[*]gilles.truan@insa-toulouse.fr

This PDF file includes

Figures S1 to S5
Tables S1 to S6
Equations S1 to S2
SI References

Supporting Information Figures

Fig. S1: Phylogenetic tree of CrtI, CrtP and PDS phytoene desaturases.
Fig. S2: Sequence alignment of the eight CrtI phytoene desaturases.
Fig. S3: Alternative representation of the CrtI phylogenetic tree shown in Figure 2.
Fig. S4: Detection of the different standards of carotenes by HPLC-UV.
Fig. S5: *In vitro* analysis of the carotenes produced in two hours by eight CrtI enzymes.

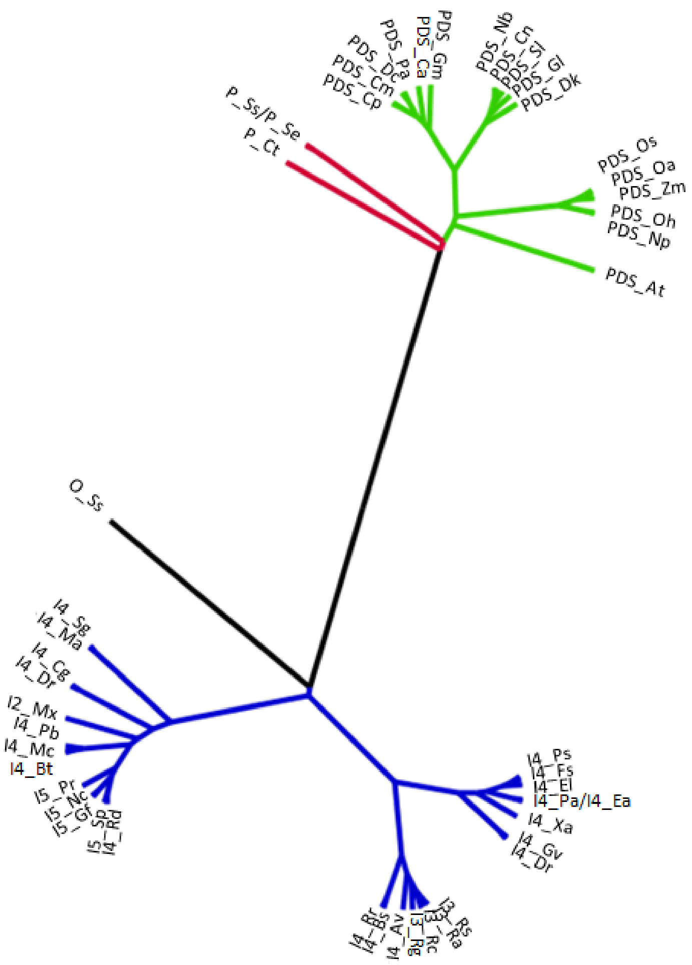

Figure S 1. Phylogenetic tree of CrtI, CrtP and PDS phytoene desaturases. Sequences extracted from Uniprot were aligned using the muscle algorithm from the SeaView suite using default parameters. The PhyML phylogenetic tree was built with the SeaView software and the following parameters: LG model, bootstrap value calculated from 100 replicates, model-given amino acid equilibrium frequencies, no invariable sites option, optimised across site rate variation and NNI operation. CrtI enzymes are in blue, CrtP in red and PDS in green. Name codes with Uniprot Id (in brackets) are as follows: I2_Mx, *Myxococcus xanthus* (P54979); I3_Rc, *Rhodobacter capsulatus* (P17054); I3_Rs, *Rhodobacter sphaeroides* (P54980); I3_Ra, *Rhodobacter azotoformans* (G3FHJ1); I4_Rg, *Rubrivivax gelatinosus* (Q9JP98); I4_Av, *Allochromatium vinosum* (D3RP90); I4_Pa, *Pantoea ananatis* (P21685); I4_Ps, *Paracoccus sp.* (P54978); I4_Bs, *Bradyrhizobium sp.* (Q7BP90); I4_El, *Erythrobacter longus* (O06757); I4_Fs, *Flavobacterium sp.* (P94790); I4_Bl, *Brevibacterium linens* (Q9KK84); I4_Ma, *Mycobacterium aurum* (Q9K566); I4_Sg, *Streptomyces griseus* (Q9EXL0); I4_Gv, *Gloeobacter violaceus* (Q7NM99); I4_Xa, *Xanthobacter autotrophicus* (Q93CI7); I4_Rr, *Rhodospirillum rubrum* (Q2RX47); I4_Bt, *Blakeslea trispora* (Q67GI0); I4_Dr, *Deinococcus radiodurans* (Q9RW08); I4_Pb, *Phycomyces blakesleeanus* (P54982); I4_Mc, *Mucor circinelloides* (Q9Y7H8); I4_Cg, *Corynebacterium glutamicum* (Q93QX5); I4_Ea, *Enterobacter agglomerans* (E9LFG2); I4_Rd, *Rhodotorula diobovata* (V5QFM4); I5_Pr, *Phaffia rhodozyma* (Q7Z858); I5_Gf, *Gibberella fujikuroi* (Q8X0Z0); I5_Nc, *Neurospora crassa* (P21334); I5_Sp, *Sporidiobolus pararoseus* (A0A0K0QVD9); P_Se, *Synechococcus elongatus* (P26294); P_Ct, *Chlorobaculum tepidum* (Q8KE83) and P_Ss, *Synechocystis sp.* (P29273); PDS_Dk, *Diospyros kaki* (D1LZY8); PDS_Cp *Citrus paradisi* (Q94KE8); PDS_Cm, *Citrus maxima* (B3VSF4); PDS_Ca, *Carica papaya* (Q0PQZ4); PDS_Pa, *Prunus armeniaca* (Q56QV3); PDS_Gl, *Gentiana lutea* (Q8VX08); PDS_Nb, *Nicotiana benthamiana* (B1NYI4); PDS_Os, *Oryza sativa subsp. japonica* (Q0DUI8); PDS_Np, *Narcissus pseudonarcissus* (Q40406); PDS_Oa, *Oryza sativa subsp. indica* (A2XDA1); PDS_Sl, *Solanum lycopersicum* (P28554); PDS_Zm, *Zea mays* (P49086); PDS_Gm, *Glycine max* (P28553); PDS_At, *Arabidopsis thaliana* (Q07356); PDS_Ca, *Capsicum annuum* (P80093); PDS_Oh, *Oncidium hybrid cultivar* (C3VEP9) and PDS_Dc, *Daucus carota subsp. sativus* (Q2VER7). O_Ss, the β-carotene ketolase from *Synechocystis sp.* (A0A068N4T0), is used as an outgroup protein to root the tree. The phylogenetic tree was generated using FigTree v1.4.3 (http://tree.bio.ed.ac.uk/software/figtree/).

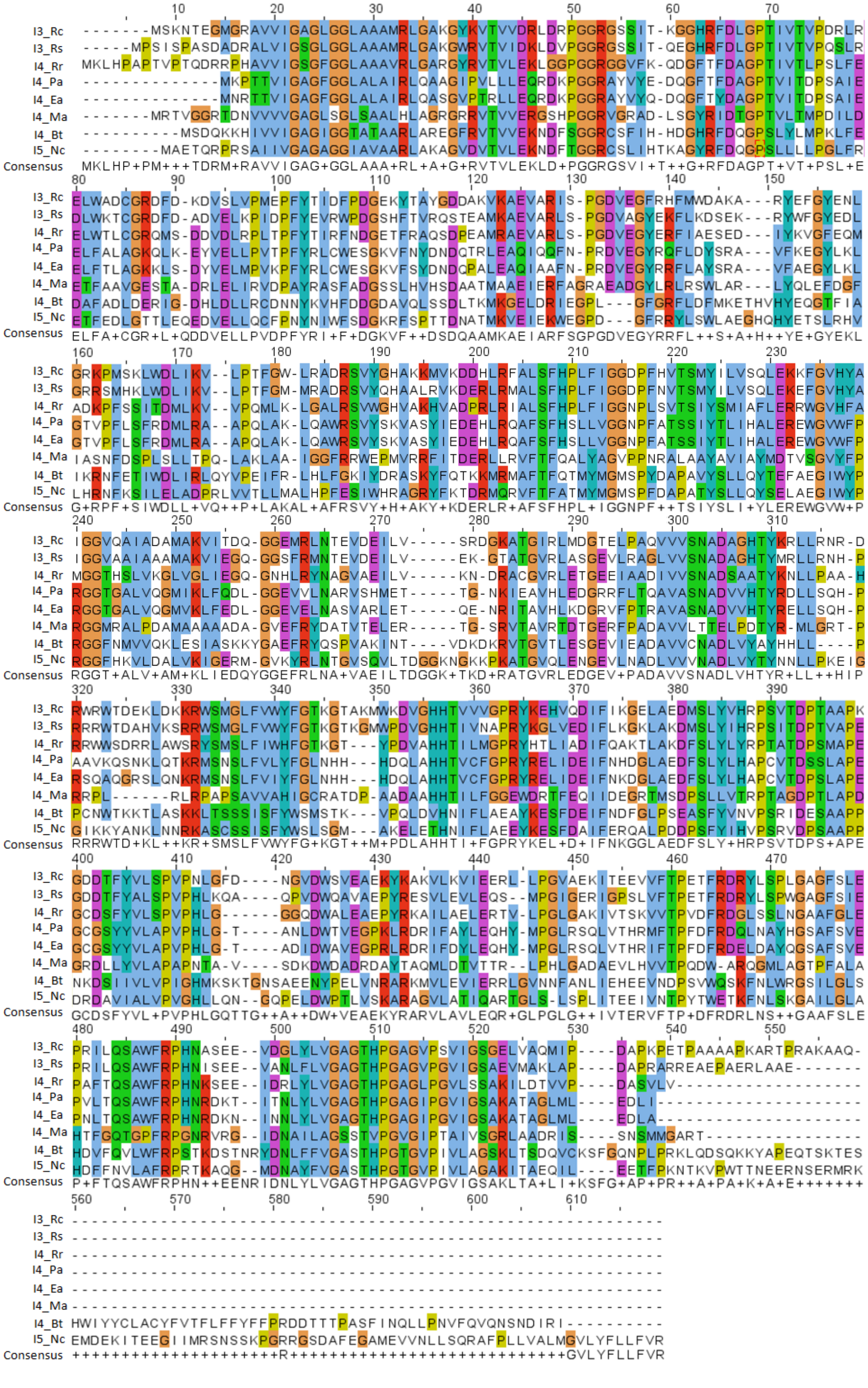

Figure S 2. Sequence alignment of the eight CrtI phytoene desaturases. CrtI codes are identical to the ones depicted in Table S2. The alignment was colour coded using the ClustalX colour scheme, visualizing simultaneously amino acid conservation and their physico-chemical properties. The alignment and consensus sequence were generated using Jalview v2.11.1.3 (http://www.jalview.org/)

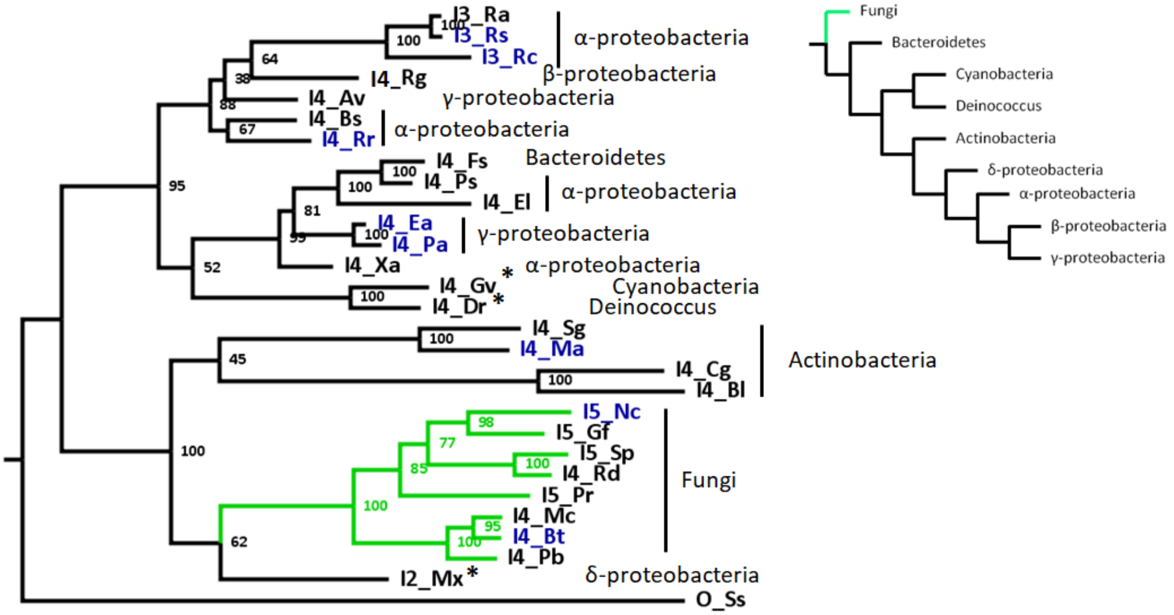

Figure S 3. Alternative representation of the CrtI phylogenetic tree shown in Figure 2. Names in blue represent enzymes used in this study. Organisms groups are presented along with the CrtI names. At the extreme right, a schematic phylogenetic tree of the different class of bacteria and fungi is presented. In cyanobacteria, the phytoene desaturation reactions are normally performed by two distinct enzymes, namely CrtP and CrtQ. I4_Gv is closely related to I4_Dr (both tagged with asterisks) and is the sole example of CrtI enzyme in cyanobacteria. Because of the high sequence identity between I4_Gv and I4_Dr, we can hypothesise that the former originated from a gene transfer from *D. radiodurans* that appeared only in the cyanobacteria *G. violaceus*. This CrtI enzyme allows *G. violaceus* to exhibit a unique pattern of carotenes in the cyanobacteria phylum[1]. I2_Mx (tagged in the tree with an asterisk), the only known 2-steps desaturase from the CrtI family, produces ζ-carotene which is further modified by a second, specific, desaturase. Hence, two desaturases activities are necessary to form lycopene, unlike all other organisms using CrtI enzymes analysed here. The phylogenetic tree was generated using FigTree v1.4.3 (http://tree.bio.ed.ac.uk/software/figtree/).

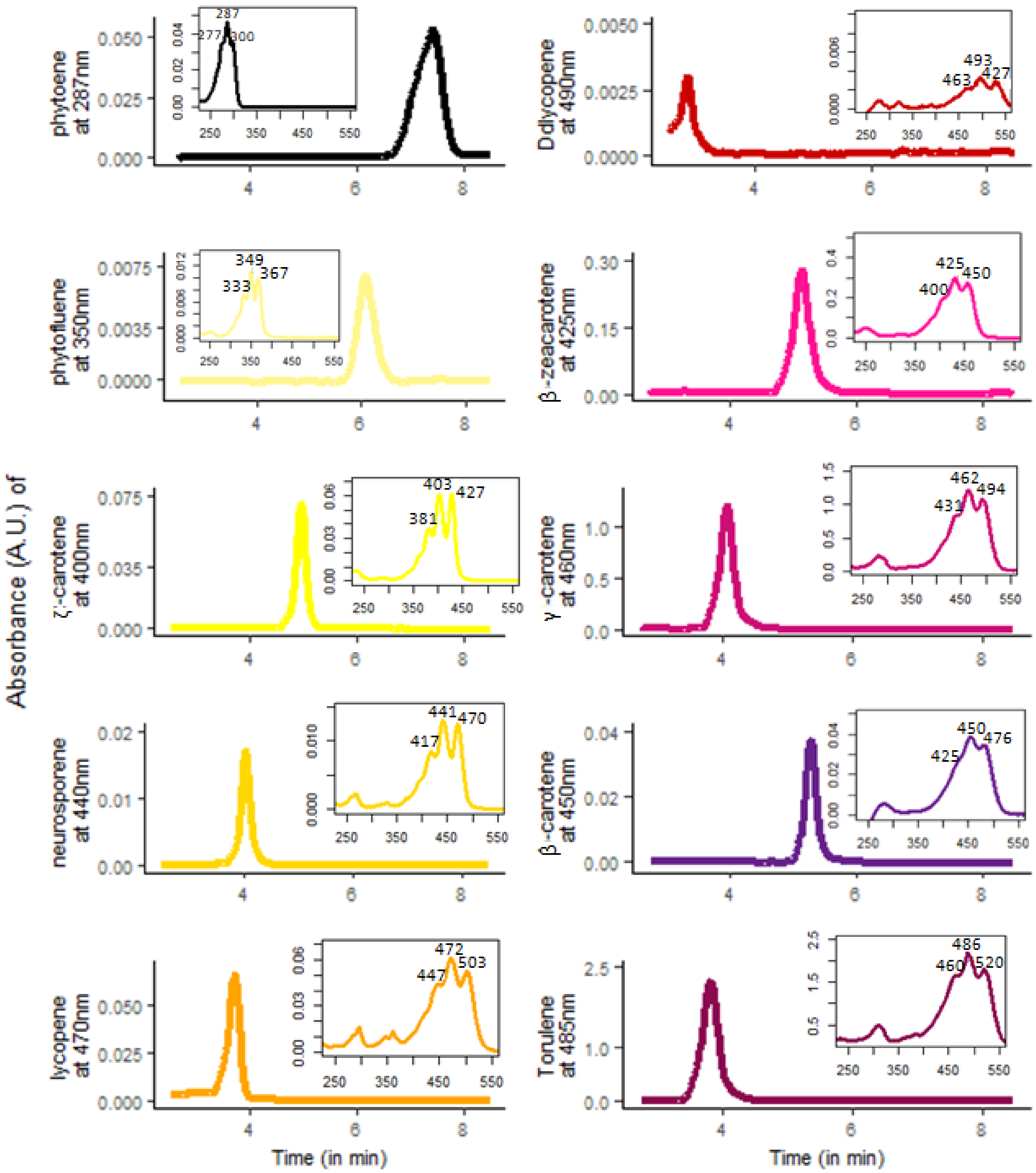

Figure S 4. Detection of the different standards of carotenes by HPLC-UV. Insets represent the full absorbance spectrum of the molecule. Graphs were generated using RStudio v1.1.463 (https://rstudio.com/). Ddlycopene = didehydrolycopene.

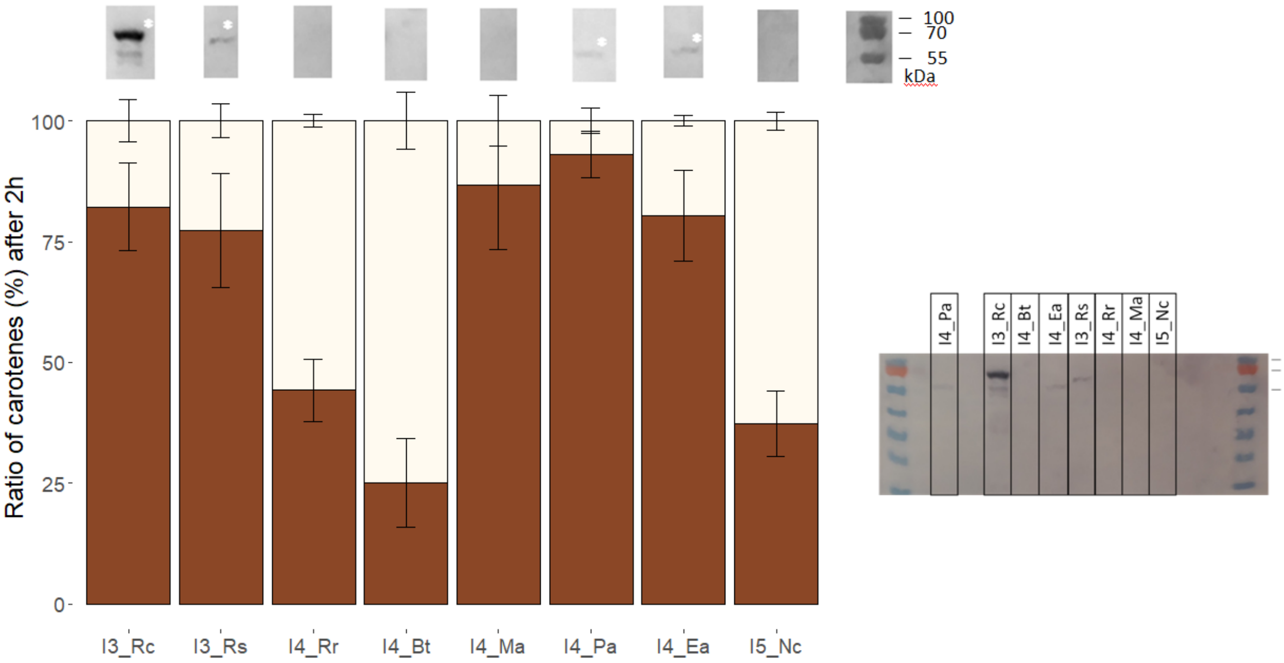

Figure S 5. *In vitro* analysis of the carotenes produced in two hours by eight CrtI enzymes. Carotenes were analysed after enzymatic assays performed on the soluble fraction of crude extracts. Top: Western Blots of the eight studied CrtI enzymes expressed in *E. coli*. CrtI enzymes were detected via a His-tag introduced at the C-termini of the sequences. Bottom: carotenes were quantified by HPLC-UV and are reported as a fraction of the total carotene content. Phytoene is represented in ivory, desaturated products in brown. Right: unprocessed version of the fulllength Western Blot. Graphs were generated using RStudio v1.1.463 (https://rstudio.com/).

Supporting Information Tables

Table S1 : Properties of carotenes analysed in the article.
Table S2 : Phytoene desaturases used in our study.
Table S3 : Proportions of the different carotenes produced in a sixty hours incubation with phytoene by five CrtI enzymes expressed in *E. coli*.
Table S4: Dissociation constants (K_d) and turnover numbers (k_{cat}) were calculated from the rate constants obtained by fitting the two hours kinetic data with the enzymatic model.
Table S5: Quantity of carotenes (in μM/10 mL culture) extracted from yeast strains producing the phytoene pathway and expressing or not the lycopene cyclase and three phytoene desaturases: I3_Rs, I4_Bt and I5_Nc.
Table S6 : Nucleic acid sequences of the codon optimized genes coding for the CrtI enzymes used in this work.

Table S 1. Properties of carotenes analysed in the article. Molar extinction coefficients are reported from Foppen, *et al.*[2] and Takaichi et *al.*[3]. Wavelengths underlined and in bold are used to characterise and to quantify studied carotenes. Retention times and absorption maxima correspond to data extracted from our HPLC-UV analysis.

Name (Provider)	CAS	Maxima of absorbance spectra (in nm)	Molar extinction coefficient (ε, in $L.mol^{-1}.cm^{-1}$)	Retention time in our study (in min)
Phytoene (Carotenature and purified in this study)	13920-14-4	277, **<u>287</u>**, 300	60,000	7.3
Phytofluene (Carotenature)	27664-65-9	333, **<u>349</u>**, 367	82,000	6.1
ζ-carotene (Carotenature)	502-63-6	381, **<u>403</u>**, 425	123,000	5.0
Neurosporene (Carotenature)	502-64-7	417, **<u>441</u>**, 470	150,000	4.1
Lycopene (Carotenature)	502-65-8	447, **<u>472</u>**, 503	185,000	3.7
Didehydro-lycopene (Purified in this study)	7797-89-9	463, **<u>493</u>**, 527	160,000	2.7
Tetradehydro-lycopene	4481-63-4	476, **<u>504</u>**, 542	/	2.4
β-zeacarotene (Carotenature)	514-90-9	400, **<u>425</u>**, 450	105,000	4.9
Torulene (Carotenature)	547-23-9	460, **<u>486</u>**, 520	173,000	3.8
7,8-dihydro-β-carotene	58218-99-8	410, **<u>429</u>**, 454	/	7.0
β-carotene (Sigma)	7235-40-7	425, **<u>450</u>**, 476	139,000	5.1
γ-carotene (Carotenature)	472-93-5	431, **<u>462</u>**, 494	165,000	4.0

Table S 2. Phytoene desaturases used in our study. Lines highlighted in grey represent the eight CrtI enzymes selected for biochemical analysis.

Name	Origin	UniProt ID	Final carotenoid product	
			In literature	In our study
I2_Mx	*Myxococcus xanthus*	P54979	ζ-carotene (natural host)[4]	
I3_Rc	*Rhodobacter capsulatus*	P17054	Neurosporene (natural host)[5]<br>Lycopene (*in vitro*)[6]	 Lycopene (*in vitro*)
I3_Rs	*Rhodobacter sphaeroides*	P54980	Neurosporene (natural host)[7]<br>Lycopene (*in vitro*)[6]	Neurosporene (*in vivo*)<br>Neurosporene (*in vitro*)
I3_Ra	*Rhodobacter azotoformans*	G3FHJ1	Neurosporene (natural host)[8]<br>Didehydrolycopene (*in vivo*)[8]	
I4_Rg	*Rubrivivax gelatinosus*	Q9JP98	Neurosporene (natural host)[9]<br>Lycopene (natural host)[10]<br>Tetradehydrolycopene (*in vitro*)[11]	
I4_Av	*Allochromatium vinosum*	D3RP90	Lycopene (natural host)[12]	
I4_Pa	*Pantoea ananatis*	P21685	Lycopene (natural host)[13]<br>Tetradehydrolycopene (*in vitro*)[14]	 Tetradehydrolycopene (*in vitro*)
I4_Ps	*Paracoccus sp.*	P54978	Lycopene (natural host)[15]<br>Lycopene (*in vivo*)[16]	
I4_Bs	*Bradyrhizobium sp.*	Q7BP90	Lycopene (natural host)[17]<br>Lycopene (*in vivo*)[17]	
I4_El	*Erythrobacter longus*	O06757	Lycopene (natural host)[18]<br>Lycopene (*in vivo*)[19]	
I4_Fs	*Flavobacterium sp.*	P94790	Lycopene (natural host)[20]<br>Lycopene (*in vivo*)[20]	
I4_Bl	*Brevibacterium linens*	Q9KK84	Lycopene (natural host)[21]<br>Didehydrolycopene (*in vitro*)[22]	
I4_Ma	*Mycobacterium aurum*	Q9K566	Lycopene (natural host)[23]<br>Lycopene (*in vivo*)[24]	 Lycopene (*in vitro*)
I4_Sg	*Streptomyces griseus*	Q9EXL0	Lycopene (natural host)[25]	
I4_Gv	*Gloeobacter violaceus*	Q7NM99	Lycopene (natural host)[1]	
I4_Xa	*Xanthobacter autotrophicus*	Q93CI7	Lycopene (natural host)[26]<br>Lycopene (*in vivo*)[26]	
I4_Rr	*Rhodospirillum rubrum*	Q2RX47	Lycopene (natural host)[27]	Lycopene (*in vitro*)
I4_Bt	*Blakeslea trispora*	Q67GI0	Lycopene (natural host)[28]<br>Lycopene (*in vitro*)[29]	Didehydrolycopene (*in vivo*)<br>Lycopene (*in vitro*)
I4_Dr	*Deinococcus radiodurans*	Q9RW08	Lycopene (natural host)[30]<br>Lycopene (*in vivo*)[30]	
I4_Pb	*Phycomyces blakesleeanus*	P54982	Lycopene (natural host)[31]<br>Lycopene (*in vivo*)[31]	
I4_Mc	*Mucor circinelloides*	Q9Y7H8	Lycopene (natural host)[32]<br>Lycopene (*in vivo*)[32]	
I4_Cg	*Corynebacterium glutamicum*	Q93QX5	Lycopene (natural host)[33]<br>Didehydrolycopene (*in vitro*)[6]	
I4_Ea	*Enterobacter agglomerans*	E9LFG2	Lycopene (natural host)[34]<br>Tetradehydrolycopene (*in vivo*)[6]	 Tetradehydrolycopene (*in vitro*)
I4_Rd	*Rhodotorula diobovata*	V5QFM4	Lycopene (natural host)[35]<br>Lycopene (*in vitro*)[36]	
I5_Pr	*Phaffia rhodozyma*	Q7Z858	Didehydrolycopene (natural host)[37]<br>Lycopene (*in vitro*)[38]	
I5_Gf	*Gibberella fujikuroi*	Q8X0Z0	Didehydrolycopene (natural host)[39]<br>Didehydrolycopene (*in vitro*)[40]	
I5_Nc	*Neurospora crassa*	P21334	Didehydrolycopene (natural host)[41]<br>Didehydrolycopene (*in vitro*)[42]	Didehydrolycopene (*in vivo*)<br>Tetradehydrolycopene (*in vitro*)
I5_Sp	*Sporidiobolus pararoseus*	A0A0K0QVD9	Didehydrolycopene (natural host)[43]<br>Didehydrolycopene (*in vivo*)[44]	

Table S 3. Proportions of the different carotenes produced in a sixty hours incubation with phytoene by five CrtI enzymes expressed in *E. coli*.

Desaturases	Carotene	Relative content (in %)
I3_Rc	Phytoene	7.8
	Phytofluene	33.8
	ζcarotene	6.5
	Neurosporene	31.3
	Lycopene	20.6
	Didehydrolycopene	0.0
	Tetradehydrolycopene	0.0
I4_Bt	Phytoene	73.4
	Phytofluene	13.6
	ζcarotene	1.8
	Neurosporene	1.9
	Lycopene	9.3
	Didehydrolycopene	0.0
	Tetradehydrolycopene	0.0
I4_Pa	Phytoene	5.9
	Phytofluene	9.2
	ζcarotene	1.1
	Neurosporene	0.2
	Lycopene	82.5
	Didehydrolycopene	0.5
	Tetradehydrolycopene	0.6
I4_Ea	Phytoene	6.9
	Phytofluene	7.5
	ζcarotene	1.7
	Neurosporene	0.3
	Lycopene	82.4
	Didehydrolycopene	0.6
	Tetradehydrolycopene	0.5
I5_Nc	Phytoene	23.0
	Phytofluene	19.0
	ζcarotene	6.5
	Neurosporene	4.5
	Lycopene	1.5
	Didehydrolycopene	41.9
	Tetradehydrolycopene	3.5

Table S 4. Dissociation constants (K_d) and turnover numbers (k_{cat}) were calculated from the rate constants obtained by fitting the two hours kinetic data with the enzymatic model. Constants are expressed in in μM (K_d) and s^{-1} (k_{cat}). ND, not determined due to the absence, in two hours, of didehydrolycopene formation from lycopene.

	Phytoene		Phytofluene		ζ-carotene		Neurosporene		Lycopene	
	K_d	k_{cat}	K_d	k_{cat}	K_d	k_{cat}	K_d	k_{cat}	K_d	k_{cat}
I3_Rc	1.1	0.003	1	0.001	23	0.04	33	$2.4x10^{-4}$	100	ND
I4_Bt	7.9	0.1	4.7	1.0	2.5	0.1	1.6	0.1	100	ND
I4_Ma	13.1	0.1	1.0	0.1	2.3	0.1	2.1	0.1	100	ND
I4_Pa	92.8	0.1	1.0	0.03	24.8	0.1	8.4	0.1	39	ND
I4_Ea	61.8	0.1	3.4	0.1	19.4	0.1	1.0	0.01	100	ND

Table S 5. Quantity of carotenes (in μM/10 mL culture) extracted from yeast strains producing the phytoene pathway and expressing or not the lycopene cyclase and three phytoene desaturases: I3_Rs, I4_Bt and I5_Nc. Numbers in parenthesis represent the percentage of phytofluene, ζ-carotene, β-zeacarotene, 7,8-dihydro-β-carotene, neurosporene, β-carotene, lycopene, torulene or didehydrolycopene compared to the total amount of carotenes formed from phytoene. γ-carotene was not included in the calculation, because the amounts detected were extremely low compared to other carotenes.

		I3_Rs				I4_Bt				I5_Nc			
		Phytoene Synthase		Phytoene Synthase + Lycopene Cyclase		Phytoene Synthase		Phytoene Synthase + Lycopene Cyclase		Phytoene Synthase		Phytoene Synthase + Lycopene Cyclase	
	Carotene	Quantity	S.D.	Quantity	S.D.	Quantity	S.D.	Quantity	S.D.	Quantity	S.D.	Quantity	S.D.
Non cyclized carotenes	Phytoene	9.5	0.3	313.8	33.5	7.9	0.3	367.4	108	4.5	1	67.8	45.2
	Phytofluene	4.4 (67.7)	0.2	27.7 (91.7)	0.4	0.7 (0.7)	0.1	134.5 (4.7)	48.7	0.2 (1.0)	0.1	45.6 (5.3)	30.4
	ζ-carotene	1.4 (21.5)	0.1	2.2 (7.3)	0.1	0.3 (0.3)	0.1	6.5 (0.2)	0.7	0.3 (1.5)	0.1	2.1 (0.2)	1.4
	Neurosporene	0.7 (10.8)	0.1	0.3 (1.0)	0.03	0.02 (<0.1)	0.01	13.4 (0.5)	5.1	0.1 (0.5)	0.02	0.9 (0.1)	0.7
	Lycopene	0	0	0	0	99.9 (98.8)	20.5	8.9 (0.3)	2.2	0	0	21.7 (2.5)	14.5
	Didehydrolycopene	0	0	0	0	0.2 (0.2)	0.1	0	0	19.6 (97.0)	2.4	10.5 (1.2)	7
Cyclized carotenes	β-zeacarotene	0	0	0.006 (<0.1)	0.007	0	0	0.1 (<0.1)	0.03	0	0	0.1 (<0.1)	0.1
	γ-carotene	0	0	0	0	0	0	0.006 (<0.1)	0.003	0	0	0.03 (<0.1)	0.02
	Torulene	0	0	0	0	0	0	0	0	0	0	0.1 (<0.1)	0.05
	7,8-dihydro-β-carotene	0	0	0	0	0	0	0	0	0	0	1.2 (0.1)	0.8
	β-carotene	0	0	0	0	0	0	2711.7 (94.3)	529	0	0	782.1 (90.5)	521.3

Table S 6. Nucleic acid sequences of the codon optimized genes coding for the CrtI enzymes used in this work.

Name	Nucleic acid sequences
I3_Rc	ATGTCCAAGAACACAGAAGGTATGGGTCGCGCCGTTGTCATCGGTGCCGGCCTGGGCGGTCTGGCT<br>GCAGCGATGCGTCTGGGCGCAAAAGGTTACAAGGTGACGGTCGTCGATCGTCTGGATCGTCCGGGC<br>GGGCGTGGCTCTTCGATCACCAAGGGCGGGCATCGTTTCGACCTGGGGCCGACGATCGTGACGGTG<br>CCGGACCGTCTGCGCGAGCTGTGGGCCGATTGCGGGCGCGATTTCGACAAGGACGTGAGCCTGGTG<br>CCGATGGAGCCGTTCTACACCATCGATTTCCCGGATGGCGAGAAATACACCGCTTACGGCGATGACG<br>CCAAGGTCAAGGCCGAGGTGGCGCGTATCAGCCCGGGCGATGTCGAGGGCTTCCGCCATTTCATGT<br>GGGACGCCAAGGCCCGTTATGAATTTGGCTATGAAAACCTCGGCCGCAAGCCGATGAGCAAGCTGT<br>GGGACCTGATCAAGGTTCTGCCGACTTTCGGCTGGCTGAGAGCAGACCGCTCGGTCTATGGCCATGC<br>CAAGAAGATGGTGAAGGACGACCACCTGCGCTTCGCGCTGTCGTTCCATCCGCTGTTCATCGGCGGC<br>GACCCGTTCCATGTGACGTCGATGTATATCCTCGTCAGCCAGCTCGAAAAGAAATTCGGCGTGCATTA<br>CGCGATCGGCGGCGTGCAGGCGATTGCCGATGCGATGGCCAAGGTGATCACCGATCAGGGCGGCGA<br>GATGCGCCTGAACACCGAGGTCGACGAGATCCTGGTCTCGCGTGACGGCAAGGCCACGGGCATCCGT<br>CTGATGGACGGCACCGAGCTGCCGGCGCAGGTTGTCGTCTCGAACGCCGATGCGGGCCACACCTACA<br>AGCGTCTCCTGCGCAACCGCGACCGCTGGCGCTGGACCGACGAGAAGCTCGACAAGAAGCGCTGGTC<br>GATGGGGCTGTTCGTCTGGTATTTCGGCACCAAGGGTACGGCCAAGATGTGGAAGGATGTGGGTCAC<br>CACACCGTCGTCGTCGGGCCGCGCTACAAGGAACATGTGCAGGACATCTTCATCAAGGGCGAGCTGG<br>CCGAGGACATGAGCCTGTATGTCCACCGTCCGTCGGTCACTGATCCGACCGCGGCGCCGAAAGGCGA<br>CGACACCTTCTACGTGCTGTCGCCGGTGCCGAACCTCGGCTTCGACAATGGCGTGGACTGGTCGGTCG<br>AGGCCGAGAAATACAAGGCCAAGGTGCTGAAAGTGATCGAGGAACGTCTGCTGCCGGGGGTTGCCG<br>AAAAGATCACCGAGGAAGTGGTCTTCACGCCGGAAACCTTCCGCGACCGTTATCTCTCGCCGCTGGGC<br>GCGGGCTTCTCGCTGGAACCGCGTATCCTGCAATCGGCCTGGTTCCGCCCGCATAACGCCTCGGAAGA<br>GGTGGACGGGCTGTATCTGGTCGGAGCTGGAACCCATCCGGGCGCCGGTGTGCCGTCGGTGATCGGT<br>TCGGGAGAGCTGGTCGCGCAGATGATCCCGGATGCGCCGAAGCCGGAGACCCCGGCGGCGGCTGCG<br>CCGAAGGCCCGTACGCCGCGTGCCAAGGCGGCGCAATGA
I3_Rs	ATGCCGTCGATCAGTCCTGCGTCCGACGCAGACCGTGCTCTTGTGATTGGATCAGGTCTG<br>GGTGGGCTTGCCGCGGCAATGCGCTTAGGAGCCAAGGGATGGCGCGTAACGGTGATTGA<br>CAAACTGGACGTTCCAGGAGGGCGCGGATCTTCAATCACCCAGGAGGGGCATCGTTTCGA<br>CTTAGGCCCAACAATTGTAACAGTGCCACAGAGTTTACGCGACCTGTGGAAAACGTGTGGT<br>CGTGACTTTGACGCCGACGTAGAACTGAAACCAATCGACCCGTTTTACGAAGTCCGCTGGC<br>CCGACGGATCACATTTCACGGTGCGCCAGAGTACAGAGGCGATGAAGGCTGAAGTCGCCC<br>GCTTATCCCCTGGAGACGTAGCTGGATATGAAAAGTTTCTGAAGGATTCGGAGAAACGCTA<br>TTGGTTTGGATACGAGGACTTGGGCCGTCGCTCAATGCACAAGTTATGGGATCTTATTAAAG<br>TTTTGCCGACGTTCGGAATGATGCGCGCCGACCGTTCGGTGTACCAGCATGCAGCTTTACGC<br>GTAAAGGATGAGCGTTTACGTATGGCGCTTAGCTTTCATCCCCTTTTTATTGGGGGCGACCC<br>GTTTAACGTTACATCCATGTATATTCTTGTCAGCCAATTGGAGAAGGAGTTCGGTGTGCATT<br>ATGCTATTGGAGGCGTAGCCGCAATTGCAGCAGCAATGGCCAAGGTCATCGAGGGACAAG<br>GAGGGAGCTTTCGCATGAATACCGAAGTGGACGAGATTTTGGTCGAAAAGGGAACTGCGA<br>CGGGTGTTCGCCTTGCCAGTGGTGAAGTTCTGCGTGCAGGCTTGGTCGTAAGCAATGCAGA<br>TGCGGGACACACCTATATGCGTTTGTTGCGTAACCATCCACGTCGCCGCTGGACGGACGCT<br>CATGTGAAGTCCCGTCGTTGGAGCATGGGTCTTTTCGTATGGTATTTCGGTACCAAGGGGA<br>CAAAGGGTATGTGGCCTGATGTCGGACATCACACTATCGTCAACGCGCCGCGTTATAAAGG<br>ATTAGTAGAAGACATTTTTCTTAAGGGTAAACTTGCCAAGGACATGAGTCTTTATATCCACCG<br>CCCATCTATCACGGACCCGACGGTAGCACCTGAGGGGGATGATACATTTTATGCACTGTCGC<br>CCGTGCCGCACCTGAAACAAGCACAGCCGGTAGACTGGCAGGCTGTGGCAGAACCTTATCG<br>TGAGAGTGTCCTTGAGGTGTTAGAGCAGAGTATGCCTGGAATTGGTGAGCGTATTGGACCA<br>TCTTTAGTCTTCACGCCTGAAACTTTCCGTGACCGCTACTTGTCGCCTTGGGGGGCAGGCTTTT<br>CTATTGAGCCTCGCATCCTTCAGTCAGCGTGGTTCCGTCCACATAACATTAGTGAAGAGGTAG<br>CGAATTTGTTTTTGGTAGGCGCGGGTACTCACCCAGGTGCGGGTGTACCGGGTGTCATCGGCT<br>CAGCCGAGGTTATGGCGAAATTAGCGCCTGATGCGCCTCGTGCCCGCCGCGAAGCGGAGCCT<br>GCGGAACGTTTAGCCGCAGAGTGA

I4_Pa	ATGAAACCAACTACGGTAATTGGTGCAGGCTTCGGTGGCCTGGCACTGGCAATTCGTCTA<br>CAAGCTGCGGGGATCCCCGTCTTACTGCTTGAACAACGTGATAAACCCGGCGGTCGGGCT<br>TATGTCTACGAGGATCAGGGGTTTACCTTTGATGCAGGCCCGACGGTTATCACCGATCCCA<br>GTGCCATTGAAGAACTGTTTGCACTGGCAGGAAAACAGTTAAAAGAGTATGTCGAACTGC<br>TGCCGGTTACGCCGTTTTACCGCCTGTGTTGGGAGTCAGGGAAGGTCTTTAATTACGATAA<br>CGATCAAACCCGGCTCGAAGCGCAGATTCAGCAGTTTAATCCCCGCGATGTCGAAGGTTAT<br>CGTCAGTTTCTGGACTATTCACGCGCGGTGTTTAAAGAAGGCTATCTAAAGCTCGGTACTG<br>TCCCTTTTTATCGTTCAGAGACATGCTTCGCGCCGCACCTCAACTGGCGAAACTGCAGGCA<br>TGGAGAAGCGTTTACAGTAAGGTTGCCAGTTACATCGAAGATGAACATCTGCGCCAGGCGT<br>TTTCTTTCCACTCGCTGTTGGTGGGCGGCAATCCCTTCGCCACCTCATCCATTTATACGTTGA<br>TACACGCGCTGGAGCGTGAGTGGGGCGTCTGGTTTCCGCGTGGCGGCACCGGCGCATTAG<br>TTCAGGGGATGATAAAGCTGTTTCAGGATCTGGGTGGCGAAGTCGTGTTAAACGCCAGAGT<br>CAGCCATATGGAAACGACAGGAAACAAGATTGAAGCCGTGCATTTAGAGGACGGTCGCAG<br>GTTCCTGACGCAAGCCGTCGCGTCAAATGCAGATGTGGTTCATACCTATCGCGACCTGTTAA<br>GCCAGCACCCTGCCGCGGTTAAGCAGTCCAACAAACTGCAGACTAAGCGCATGAGTAACTCT<br>CTGTTTGTGCTCTATTTTGGTTTGAATCACCATCATGATCAGCTCGCGCATCACACGGTTTGTT<br>TCGGCCCGCGTTACCGCGAGCTGATTGACGAAATTTTTAATCATGATGGCCTCGCAGAGGAC<br>TTCTCACTTTATCTGCACGCGCCCTGTGTCACGGATTCGTCACTGGCGCCTGAAGGTTGCGGC<br>AGTTACTATGTGTTGGCGCCGGTGCCGCATTTAGGCACCGCGAACCTCGACTGGACGGTTGA<br>GGGGCCAAAACTACGCGACCGTATTTTTGCGTACCTTGAGCAGCATTACATGCCTGGCTTACG<br>GAGTCAGCTGGTCACGCACCGGATGTTTACGCCGTTTGATTTTCGCGACCAGCTTAATGCCTA<br>TCATGGCTCAGCCTTTTCTGTGGAGCCCGTTCTTACCCAGAGCGCCTGGTTTCGGCCGCATAA<br>CCGCGATAAAACCATTACTAATCTCTACCTGGTCGGCGCAGGCACGCATCCCGGCGCAGGCAT<br>TCCTGGCGTCATCGGCTCGGCAAAAGCGACAGCAGGTTTGATGCTGGAGGATCTGATATGA
I4_Ma	ATGCGTACGGTGGGAGGTCGCACCGACAACGTCGTAGTAGTAGGCGCCGGGCTTTCGGGT<br>TTATCAGCGGCGTTACACTTAGCTGGACGCGGGCGCCGCGTCACGGTTGTAGAACGTGGATCGCACC<br>CAGGCGGCCGTGTAGGACGTGCGGATCTTAGTGGGTATCGCATCGATACTGGACCCACGGTCTTAAC<br>TATGCCTGATATTCTTGATGAGACGTTCGCGGCTGTAGGCGAGTCTACCGCTGATCGCTTAGAATTGA<br>TCCGTGTGGACCCAGCATATCGTGCATCCTTTGCCGACGGCAGTTCACTTCACGTCCATTCCGACGCG<br>GCAACGATGGCTGCCGAAATTGAACGCTTTGCTGGGCGTGCGGAGGCTGACGGTTATTTACGTTTGC<br>GTTCGTGGTTGGCCCGCCTTTACCAATTGGAGTTTGACGGATTTATCGCTTCAAACTTCGATTCCCCAC<br>TGAGCCTGCTGACACCCCAGCTTGCGAAACTTGCAGCGATTGGAGGGTTTCGCCGCTGGGAACCCAT<br>GGTCCGCCGTTTTATTACTGATGAACGTCTTTTGCGTGTCTTTACCTTCCAAGCCCTTTACGCGGGGGT<br>GCCCCCAAACCGTGCGTTAGCTGCGTATGCGGTAATCGCATATATGGACACGGTGAGCGGCGTATAC<br>TTTCCCCGTGGCGGTATGCGCGCACTGCCCGACGCCATGGCCGCCGCTGCTGCTGACGCAGGCGTTG<br>AGTTCCGCTACGATGCTACTGTCACCGAGCTGGAACGCACCGGCTCCCGTGTTACAGCTGTGCGCAC<br>AGATACCGGTGAACGCTTTCCGGCCGATGCCGTAGTTCTTACAACGGAGTTGCCGGATACATATCGC<br>ATGCTGGGCCGTACACCCCGCCGTCCTTTACGTCTTCGTCCAGCGCCTAGCGCCGTAGTGGCGCATAT<br>TGGGTGCCGTGCCACGGATCCCGCAGCCGACGCCGCTCATCATACTATTCTGTTTGGTGGGGAGTGG<br>GATCGCACATTCGAACAAATTATCGATGAAGGTCGTACGATGTCCGATCCCTCATTGTTGGTTACCCG<br>TCCCACAGCCGGTGATCCCACCTTGGCGCCAGATGGGCGTGACTTGCTGTACGTCTTGGCCCCGGCC<br>CCTAATACCGCCGTATCGGATAAAGACTGGGACGCCGACCGCGACGCCTATACAGCGCAAATGCTG<br>GATACTGTAACAACGCGCTTGCCGCACTTGGGAGCCGACGCCGAAGTCCTTCACGTTGTAACACCAC<br>AGGACTGGGCACGCCAGGGTATGTTAGCAGGTACGCCATTCGCCCTGGCTCACACTTTCGGGCAGA<br>CAGGACCTTTTCGCCCTGGCAACCGCGTTCGCGGAATCGATAACGCTATCTTAGCAGGCTCGTCCAC<br>CGTTCCCGGAGTTGGGATCCCAACGGCGATCGTCTCTGGCCGCCTTGCGGCGGACCGCATCTCCAG<br>CAACAGCATGATGGGCGCGCGCACCTGA

I4_Rr	ATGAAGTTGCATCCCGCTCCAACTGTCCCGACACAAGATCGTCGCCCGCATGCTGTCGTT<br>ATCGGGTCCGGGTTTGGGGGGCTGGCGGCTGCCGTACGTCTGGGTGCCCGCGGCTACCGCGTTA<br>CCGTTCTGGAAAAACTGGGTGGTCCAGGAGGGCGTGGAGGAGTCTTCAAGCAGGACGGGTTCAC<br>GTTCGACGCTGGCCCCACGATCGTCACGTTACCATCCTTGTTCGAGGAACTTTGGACTCTTTGTGGT<br>CGCCAGATGAGTGACGATGTCGACTTGCGCCCCCTTACTCCTTTTTATACCATCCGCTTCAACGATG<br>GCGAGACATTCCGCGCCCAGAGCGATCCAGAGGCCATGCGTGCTGAAGTCGCACGCTTATCTCCTG<br>GCGACGTGGAGGGCTACGAGCGTTTCATCGCGGAATCCGAGGACATCTATAAAGTAGGATTCGA<br>ACAAATGGCGGATAAACCATTTTCCTCCATCACTGACATGCTGAAGGTCGTTCCTCAGATGCTGAA<br>ATTAGGCGCGTTACGTAGCGTGTGGGGTCATGTCGCTAAGCACGTTGCAGATCCGCGCTTGCGTA<br>TTGCCCTGAGCTTCCACCCACTTTTCATTGGTGGTAATCCCCTTTCAGTAACCTCGATCTATAGTATG<br>ATTGCATTTCTGGAACGTCGTTGGGGCGTTCATTTTGCTATGGGTGGCACGCACTCCTTGGTAAAA<br>GGTCTTGTGGGACTGATTGAGGGCCAGGGAAATCACCTTCGCTATAATGCAGGTGTAGCTGAAAT<br>CTTAGTCAAGAATGACCGTGCTTGCGGCGTACGTCTTGAAACGGGGGAAGAGATTGCCGCGGAT<br>ATCGTTGTGTCCAATGCTGACTCCGCAGCTACCTATAAGAATTTATTGCCAGCTGCGCATCGTCGCT<br>GGTGGTCCGATCGCCGTCTGGCATGGAGTCGCTACAGTATGTCTTTGTTCATTTGGCATTTCGGG<br>ACGAAGGGAACTTACCCAGACGTCGCGCACCATACGATCCTTATGGGACCCCGTTACCACACCTTG<br>ATTGCTGACATTTTCCAAGCTAAAACATTGGCTAAGGACTTTTCATTATATTTGTACCGCCCTACGG<br>CGACGGACCCCAGTATGGCCCCAGAGGGATGTGACTCTTTCTACGTCTTGTCCCCAGTGCCCCACT<br>TAGGAGGGGGGCAGGATTGGGCGCTGGAAGCCGAGCCATACCGCAAGGCCATCTTGGCGGAAC<br>TGGAACGCACGGTCTTGCCAGGGCTTGGAGCGAAAATTGTAACCAGCAAGGTAGTAACCCCTGTC<br>GACTTCCGTGATGGCCTGTCTTCTTTGAACGGGGCTGCCTTTGGGTTAGAGCCCGCGTTTACGCAA<br>TCGGCATGGTTCCGCCCCCATAATAAGAGCGAGGAGATCGACCGTTTATATTTAGTAGGCGCTGG<br>AACTCACCCAGGAGCGGGTCTGCCGGGCGTGCTGTCCTCAGCCAAGATTCTTGACACCGTCGTCCC<br>AGATGCATCTGTTCTTGTGTGA
I4_Bt	ATGTCTGATCAGAAGAAGCACATTGTCGTCATCGGTGCTGGTATCGGTGGTACTGCAACA<br>GCAGCACGTCTGGCACGTGAGGGTTTCCGTGTCACTGTCGTCGAGAAGAACGACTTCTCTGGTGG<br>TCGTTGCTCTTTCATTCACCACGACGGTCACCGTTTCGACCAGGGTCCTTCATTGTACTTGATGCCTA<br>AGTTGTTTGAGGACGCTTTCGCTGACCTGGACGAGCGTATCGGTGACCACTTGGACCTGCTGCGTT<br>GTGACAACAATTACAAAGTCCATTTCGACGACGGTGACGCTGTCCAATTGTCATCAGACCTGACAA<br>AGATGAAGGGTGAGTTGGACCGTATTGAGGGTCCTCTGGGTTTCGGTCGTTTCCTGGATTTCATGA<br>AAGAGACACACGTCCACTACGAGCAGGGTACATTCATTGCTATCAAGCGTAACTTCGAAACTATCTG<br>GGACCTGATCCGTCTGCAGTACGTCCCAGAGATTTTTCGTTTGCACCTGTTCGGTAAGATCTACGAC<br>CGTGCATCAAAATACTTCCAAACAAAAAAGATGCGTATGGCTTTTACTTTTCAAACAATGTACATGGG<br>TATGTCACCTTACGACGCACCTGCAGTCTACTCATTGTTGCAATATACAGAGTTCGCAGAGGGTATTTG<br>GTACCCACGTGGTGGTTTCAACATGGTCGTCCAAAAGTTGGAGTCTATCGCTTCTAAGAAGTACGGTG<br>CTGAGTTCCGTTACCAATCTCCTGTCGCTAAGATTAACACTGTCGATAAAGACAAGCGTGTCACTGGT<br>GTCACTTTGGAGTCTGGTGAAGTCATTGAGGCAGACGCTGTCGTCTGCAACGCTGACTTGGTCTACGC<br>TTACCACCACTTGTTGCCACCTTGCAACTGGACAAAGAAGACTTTGGCATCTAAGAAACTGACATCTTC<br>ATCAATTTCTTTTTACTGGTCAATGTCTACTAAGGTCCCTCAATTGGACGTCCACAACATTTTCTTGGCT<br>GAGGCTTACAAGGAGTCATTCGACGAGATTTTTAACGATTTCGGTTTGCCTTCTGAAGCATCTTTCTAC<br>GTCAACGTTCCTTCACGTATCGACGAGTCTGCAGCACCTCCAAATAAGGACTCAATTATCGTTCTGGTT<br>CCAATTGGTCACATGAAGTCTAAGACAGGTAACTCAGCAGAGGAGAACTACCCAGAGTTGGTCAACC<br>GTGCTCGTAAGATGGTCTTGGAGGTCATCGAGCGTCGTTTGGGTGTCAACAACTTCGCTAACTTGATC<br>GAACACGAGGAGGTCAACGACCCATCAGTCTGGCAATCTAAGTTCAACTTGTGGCGTGGTTCAATCCT<br>GGGTCTGTCACATGATGTCTTTCAGGTTTTGTGGTTCCGTCCTTCAACAAAGGACTCTACTAACCGTTAT<br>GACAATCTGTTTTTCGTCGGTGCATCAACTCACCCTGGTACAGGTGTCCCAATCGTCTTGGCAGGTTCT<br>AAACTGACTTCTGACCAGGTCTGTAAGTCATTCGGTCAAAACCCTTTGCCTCGTAAGCTGCAGGACTCT<br>CAGAAGAAATATGCACCTGAGCAAACACGTAAGACTGAGTCACACTGGATTTATTACTGCCTGGCATG<br>CTACTTTGTCACTTTCTTGTTCTTCTATTTCTTTCCTCGTGACGACACTACTACTCCAGCATCTTTTATTAA<br>TCAGTTGTTGCCAAACGTCTTCCAAGGTCAGAACTCTAACGATATTCGTATCTAA

I4_Ea	ATGAATAGAACTACAGTAATTGGCGCAGGCTTTGGTGGTCTGGCTCTGGCCAT<br>TCGCCTTCAGGCGTCAGGCGTTCCCACCCGACTGCTGGAGCAGCGTGACAAGCCGGGCG<br>GCCGGGCTTATGTCTATCAGGATCAGGGCTTCACGTTTGATGCCGGCCCCACGGTAATCA<br>CCGATCCCAGCGCCATTGAAGAGCTGTTCACTCTGGCGGGTAAAAAGCTCTCTGACTATG<br>TCGAGCTGATGCCGGTGAAGCCGTTTTATCGCCTCTGCTGGGAGTCCGGCAAGGTGTTCA<br>GTTATGACAACGATCAGCCCGCGCTGGAAGCGCAGATTGCCGCATTTAATCCGCGTGACG<br>TTGAAGGATATCGGCGCTTTCTGGCCTATTCCCGAGCGGTGTTTGCTGAAGGCTATCTGAA<br>GCTTGGCACCGTGCCGTTTCTGTCATTCCGCGACATGCTGCGGGCCGCGCCTCAGCTGGCA<br>AAACTTCAGGCATGGCGCAGCGTTTACAGCAAAGTGGCGAGCTACATTGAAGATGAGCAT<br>CTGCGTCAGGCCTTCTCTTTCCACTCACTGCTGGTGGGCGGAAATCCGTTTGCCACTTCCTCA<br>ATCTATACCCTGATTCATGCGCTGGAACGTGAATGGGGCGTCTGGTTCCCGCGCGGTGGC<br>ACGGGCGCGCTGGTGCAGGGCATGGTGAAACTGTTTGAAGATCTGGGCGGCGAAGTGGA<br>GCTCAATGCCAGCGTTGCCCGGCTGGAGACCCAGGAAAACAGGATTACCGCGGTGCACCT<br>GAAAGATGGCCGGGTCTTCCCGACCCGCGCGGTTGCCTCCAACGCAGATGTGGTTCACACC<br>TACCGCGAACTGCTGAGCCAGCACCCCGCTTCGCAGGCGCAGGGACGGTCACTGCAGAAC<br>AAACGCATGAGTAACTCGCTGTTTGTGATCTATTTTGGCCTGAATCATCATCACGATCAGCTG<br>GCGCACCACACGGTCTGCTTTGGTCCGCGCTATCGTGAGTTGATTGATGAAATCTTTAACAA<br>AGATGGCCTGGCAGAGGACTTCTCGCTCTATCTGCATGCGCCCTGCGTGACCGATCCCTCAC<br>TGGCACCGGAAGGCTGCGGCAGCTACTACGTGCTGGCGCCGGTACCGCACCTCGGCACCGC<br>TGATATCGACTGGGCCGTTGAAGGTCCGCGCCTGCGCGATCGCATTTTCGACTATCTGGAAC<br>AGCATTACATGCCGGGCCTGCGTAGCCAGTTGGTCACGCATCGCATCTTCACGCCGTTTGATT<br>TCCGCGATGAGCTGAATGCGTATCAGGGCTCGGCCTTCTCAGTGGAGCCGATCCTGACGCAA<br>AGCGCCTGGTTCCGGCCTCACAACCGCGATAAAAATATTAATAATCTCTATCTGGTCGGTGCT<br>GGTACCCATCCTGGCGCGGGTATTCCAGGGGTGATTGGCTCGGCCAAGGCTACCGCAGGATT<br>GATGCTGGAGGATCTGGCTTGA
I5_Nc	ATGGCGGAGACTCAGCGTCCACGCAGTGCGATCATCGTCGGTGCAGGTGCAGGGGGT<br>ATCGCAGTAGCAGCCCGCCTTGCGAAAGCCGGCGTGGACGTTACAGTGTTGGAGAAGAATGAT<br>TTCACGGGAGGACGTTGTTCCTTAATCCACACCAAAGCAGGTTACCGCTTCGACCAGGGTCCATC<br>ACTTTTACTGTTACCTGGGCTGTTTCGTGAGACCTTCGAGGATTTAGGGACTACGCTTGAGCAGG<br>AGGATGTAGAGCTGTTACAATGCTTCCCTAACTACAACATCTGGTTCTCAGACGGCAAACGTTTC<br>TCGCCGACTACTGATAACGCAACAATGAAGGTAGAAATTGAAAAATGGGAAGGTCCAGACGGG<br>TTCCGCCGCTACTTATCATGGCTGGCTGAGGGGCATCAACACTACGAAACTTCCTTGCGTCATGT<br>GTTGCACCGCAACTTCAAAAGCATTTTGGAGCTTGCGGACCCGCGCCTTGTAGTCACCTTGCTGA<br>TGGCGCTTCACCCATTTGAAAGTATCTGGCATCGTGCCGGACGCTACTTTAAGACAGATCGTATG<br>CAGCGCGTCTTTACTTTCGCAACAATGTATATGGGCATGTCTCCGTTCGATGCTCCGGCAACCTAC<br>AGTTTATTACAATATTCAGAGCTTGCGGAGGGAATCTGGTATCCGCGTGGGGGTTTCCACAAGG<br>TGTTAGATGCTTTGGTTAAGATCGGCGAACGTATGGGTGTAAAATACCGCCTTAACACCGGCGTG<br>TCTCAGGTACTTACAGATGGTGGCAAGAATGGAAAGAAACCGAAAGCTACGGGCGTTCAGCTGG<br>AGAATGGCGAAGTCCTGAACGCAGACTTGGTTGTCGTGAATGCTGACCTTGTTTATACCTACAAC<br>AACCTGTTGCCTAAAGAAATTGGTGGCATCAAGAAGTATGCAAATAAATTAAACAACCGCAAAGC<br>GTCGTGCTCAAGTATCTCCTTTTACTGGTCCTTATCGGGAATGGCTAAAGAGTTGGAAACCCATAA<br>TATCTTTTTAGCGGAGGAATATAAGGAGAGTTTTGACGCTATTTTTGAGCGTCAGGCTTTACCAGA<br>CGACCCCTCCTTCTACATTCATGTACCCAGCCGTGTCGATCCGTCGGCCGCTCCGCCAGACCGCGA<br>CGCGGTAATCGCCCTTGTTCCCGTAGGCCACCTGCTTCAAAACGGCCAGCCCGAGCTGGACTGGCC<br>GACCTTAGTGTCCAAAGCCCGCGCTGGGGTATTGGCTACAATTCAAGCGCGCACCGGGCTGTCTTT<br>GTCACCTTTAATCACGGAGGAAATCGTTAACACCCCCTACACTTGGGAAACCAAGTTTAACCTGAGC<br>AAAGGGGCCATTCTGGGGTTGGCGCATGACTTTTTTAATGTCTTAGCCTTTCGTCCCCGCACTAAAG<br>CTCAGGGTATGGACAACGCTTATTTCGTCGGAGCCTCGACTCACCCGGGGACCGGTGTGCCGATTG<br>TTTTAGCAGGAGCAAAGATCACAGCCGAGCAGATCCTTGAAGAGACTTTTCCGAAAAATACCAAGG<br>TACCTTGGACTACAAATGAGGAGCGTAACTCGGAACGTATGCGTAAGGAGATGGACGAGAAAATC<br>ACAGAAGAGGGTATTATTATGCGCTCCAACTCAAGTAAGCCGGGTCGTCGTGGATCGGACGCGTTT<br>GAGGGAGCCATGGAAGTGGTTAACCTGTTAAGTCAGCGTGCGTTTCCTTTATTGGTCGCTTTAATGG<br>GGGTGTTATATTTCCTTCTGTTTGTTCGTTGA

Supplementary Information Equations

Equation S1: Equation of the phytoene desaturase CrtI reactions used in our COPASI models.
Equation S2: Differential equations generated by our COPASI model.

Equation S1: Equation of the phytoene desaturase CrtI reactions used in our COPASI models.

$$
\begin{array}{lccc}
 & CrtIPh & \underset{R_1k_f}{\overset{R_1k_r}{\rightleftarrows}} & CrtI + Ph \\
R_1k_{cat} & \downarrow & & \\
 & CrtIPf & \underset{R_2k_f}{\overset{R_2k_r}{\rightleftarrows}} & CrtI + Pf \\
R_2k_{cat} & \downarrow & & \\
 & CrtIZc & \underset{R_3k_f}{\overset{R_3k_r}{\rightleftarrows}} & CrtI + Zc \\
R_3k_{cat} & \downarrow & & \\
 & CrtINs & \underset{R_4k_f}{\overset{R_4k_r}{\rightleftarrows}} & CrtI + Ns \\
R_4k_{cat} & \downarrow & & \\
 & CrtILy & \underset{R_5k_f}{\overset{R_5k_r}{\rightleftarrows}} & CrtI + Ly
\end{array}
$$

Equation S2: Differential equations generated by our COPASI model.

$$
\begin{aligned}
\frac{d([CrtI]\times V_{tube})}{dt} = & -V_{tube}\times((0.161812\times[CrtI]\times[Ph]-9.99996\times[CrtIPh]) \\
& -V_{tube}\times(0.291607\times[CrtI]\times[Pf]-1\times[CrtIPf]) \\
& -V_{tube}\times(0.521829\times[CrtI]\times[Zc]-10\times[CrtIZc]) \\
& -V_{tube}\times(1\times[CrtI]\times[Ns]-1\times[CrtINs]) \\
& -V_{tube}\times(0.1\times[CrtI]\times[Ly]-10\times[CrtILy]) \\
\frac{d([CrtIPh]\times V_{tube})}{dt} = & -V_{tube}\times(0.1\times[CrtIPh]) \\
& +V_{tube}\times(0.161812\times[CrtI]\times[Ph]-9.99996\times[CrtIPh]) \\
\frac{d([Ph]\times V_{tube})}{dt} = & -V_{tube}\times(0.161812\times[CrtI]\times[Ph]-9.99996\times[CrtIPh]) \\
\frac{d([CrtIPf]\times V_{tube})}{dt} = & +V_{tube}\times(0.1\times[CrtIPh]) \\
& -V_{tube}\times(0.1\times[CrtIPf]) \\
& +V_{tube}\times(0.291607\times[CrtI]\times[Pf]-1\times[CrtIPf]) \\
\frac{d([Pf]\times V_{tube})}{dt} = & -V_{tube}\times(0.291607\times[CrtI]\times[Pf]-1\times[CrtIPf]) \\
\frac{d([CrtIZc]\times V_{tube})}{dt} = & +V_{tube}\times(0.1\times[CrtIPf]) \\
& -V_{tube}\times(0.1\times[CrtIZc]) \\
& +V_{tube}\times(0.521829\times[CrtI]\times[Zc]-10\times[CrtIZc]) \\
\frac{d([Pf]\times V_{tube})}{dt} = & -V_{tube}\times(0.521829\times[CrtI]\times[Zc]-10\times[CrtIZc]) \\
\frac{d([CrtINs]\times V_{tube})}{dt} = & +V_{tube}\times(0.1\times[CrtIZc]) \\
& -V_{tube}\times(0.0102255\times[CrtINs]) \\
& +V_{tube}\times(1\times[CrtI]\times[Ns]-1\times[CrtINs]) \\
\frac{d([Ns]\times V_{tube})}{dt} = & -V_{tube}\times(1\times[CrtI]\times[Ns]-1\times[CrtINs]) \\
\frac{d([CrtILy]\times V_{tube})}{dt} = & +V_{tube}\times(0.0102255\times[CrtINs]) \\
& +V_{tube}\times(0.1\times[CrtI]\times[Ly]-10\times[CrtILy]) \\
\frac{d([Ns]\times V_{tube})}{dt} = & -V_{tube}\times(0.1\times[CrtI]\times[Ly]-10\times[CrtILy])
\end{aligned}
$$

References

1. Steiger, S., Jackisch, Y. & Sandmann, G. Carotenoid biosynthesis in *Gloeobacter violaceus* PCC4721 involves a single crtI-type phytoene desaturase instead of typical cyanobacterial enzymes. *Arch. Microbiol.* **184**, 207–214 (2005).
2. Foppen, F. H. Tables for the identification of carotenoid pigments. *Chromatogr. Rev.* **14**, 133–298 (1971).
3. Takaichi, S. Characterization of carotenes in a combination of a C18 HPLC column with isocratic elution and absorption spectra with a photodiode-array detector. *Photosynth. Res.* **65**, 93 (2000).
4. Iniesta, A. A., Cervantes, M. & Murillo, F. J. Cooperation of two carotene desaturases in the production of lycopene in *Myxococcus xanthus*. *The FEBS J.* **274**, 4306–4314 (2007).
5. Raisig, A., Bartley, G., Scolnik, P. & Sandmann, G. Purification in an active state and properties of the 3-step phytoene desaturase from *Rhodobacter capsulatus* overexpressed in *Escherichia coli*. *The J. Biochem.* **119**, 559–564 (1996).
6. Song, G. H., Kim, S. H., Choi, B. H., Han, S. J. & Lee, P. C. Heterologous carotenoid-biosynthetic enzymes: functional complementation and effects on carotenoid profiles in *Escherichia coli*. *Appl. Environ. Microbiol.* **79**, 610–618 (2013).
7. Wang, C.-w. & Liao, J. C. Alteration of product specificity of *Rhodobacter sphaeroides* phytoene desaturase by directed evolution. *J. Biol. Chem.* **276**, 41161–41164 (2001).
8. Zhang, J., Lu, L., Yin, L., Xie, S. & Xiao, M. Carotenogenesis gene cluster and phytoene desaturase catalyzing both three-and four-step desaturations from *Rhodobacter azotoformans*. *FEMS Microbiol. Lett.* **333**, 138–145 (2012).
9. Steier, S., Astier, C. & Sandmann, G. Substrate specificity of the expressed carotenoid 3, 4-desaturase from *Rubrivivax gelatinosus* reveals the detailed reaction sequence to spheroidene and spirilloxanthin. *Biochem. J.* **349**, 635–640 (2000).
10. Harada, J. *et al.* Phytoene desaturase, CrtI, of the purple photosynthetic bacterium, *Rubrivivax gelatinosus*, produces both neurosporene and lycopene. *Plant Cell Physiol.* **42**, 1112–1118 (2001).
11. Stickforth, P. & Sandmann, G. Structural and kinetics properties of a mutated phytoene desaturase from *Rubrivivax gelatinosus* with modified product specificity. *Arch. Biochem. Biophys.* **505**, 118–122 (2011).
12. Bol'shakov, M., Ashikhmin, A., Makhneva, Z. & Moskalenko, A. Effect of illumination intensity and inhibition of carotenoid biosynthesis on assembly of peripheral light-gathering complexes in purple sulfur bacteria *Allochromatium vinosum* ATCC 17899. *Mikrobiologiia* **85**, 403–414 (2016).
13. Misawa, N. *et al.* Elucidation of the *Erwinia uredovora* carotenoid biosynthetic pathway by functional analysis of gene products expressed in *Escherichia coli*. *J. Bacteriol.* **172**, 6704–6712 (1990).
14. Schaub, P. *et al.* On the structure and function of the phytoene desaturase CrtI from *Pantoea ananatis*, a membrane-peripheral and FAD-dependent oxidase/isomerase. *PLoS One* **7**, e39550 (2012).
15. Yokoyama, A., Izumida, H. & Miki, W. Production of astaxanthin and 4-ketozeaxanthin by the marine bacterium, *Agrobacterium aurantiacum*. *Biosci. Biotechnol. Biochem.* **58**, 1842–1844 (1994).
16. Misawa, N. *et al.* Structure and functional analysis of a marine bacterial carotenoid biosynthesis gene cluster and astaxanthin biosynthetic pathway proposed at the gene level. *J. Bacteriol.* **177**, 6575–6584 (1995).
17. Hannibal, L. *et al.* Isolation and characterization of canthaxanthin biosynthesis genes from the photosynthetic bacterium *Bradyrhizobium sp.* strain ORS278. *J. Bacteriol.* **182**, 3850–3853 (2000).
18. Takaichi, S., Shimada, K. & Ishidsu, J.-i. Carotenoids from the aerobic photosynthetic bacterium, *Erythrobacter longus*: β-carotene and its hydroxyl derivatives. *Arch. Microbiol.* **153**, 118–122 (1990).
19. Matsumura, H., Takeyama, H., Kusakabe, E., Burgess, J. G. & Matsunaga, T. Cloning, sequencing and expressing the carotenoid biosynthesis genes, lycopene cyclase and phytoene desaturase, from the aerobic photosynthetic bacterium *Erythrobacter longus sp.* strain Och101 in *Escherichia coli*. *Gene* **189**, 169–174 (1997).
20. Pasamontes, L. *et al.* Isolation and characterization of the carotenoid biosynthesis genes of *Flavobacterium sp.* strain R1534. *Gene* **185**, 35–41 (1997).
21. Krubasik, P. & Sandmann, G. A carotenogenic gene cluster from *Brevibacterium linens* with novel lycopene cyclase genes involved in the synthesis of aromatic carotenoids. *Mol. Gen. Genet. MGG* **263**, 423–432 (2000).
22. Kim, S. H., Park, Y. H., Schmidt-Dannert, C. & Lee, P. C. Redesign, reconstruction, and directed extension of the *Brevibacterium linens* C40 carotenoid pathway in *Escherichia coli*. *Appl. Environ. Microbiol.* **76**, 5199–5206 (2010).
23. Viveiros, M., Krubasik, P., Sandmann, G. & Houssaini-Iraqui, M. Structural and functional analysis of the gene cluster encoding carotenoid biosynthesis in *Mycobacterium aurum* A+. *FEMS Microbiol. Lett.* **187**, 95–101 (2000).

24. Houssaini-Iraqui, M., Khamlichi, N., El Yamani, J. & Rastogi, N. Response of *Escherichia coli* containing mycobacterial carotene genes to UV radiation. *BioMed Res. Int.* **1**, 79–84 (2001).

25. Krügel, H., Krubasik, P., Weber, K., Saluz, H. P. & Sandmann, G. Functional analysis of genes from *Streptomyces griseus* involved in the synthesis of isorenieratene, a carotenoid with aromatic end groups, revealed a novel type of carotenoid desaturase. *Biochimica et Biophys. Acta (BBA)-Molecular Cell Biol. Lipids* **1439**, 57–64 (1999).

26. Larsen, R. A., Wilson, M. M., Guss, A. M. & Metcalf, W. W. Genetic analysis of pigment biosynthesis in *Xanthobacter autotrophicus* Py2 using a new, highly efficient transposon mutagenesis system that is functional in a wide variety of bacteria. *Arch. Microbiol.* **178**, 193–201 (2002).

27. Davies, B. A novel sequence for phytoene dehydrogenation in *Rhodospirillum rubrum*. *Biochem. J.* **116**, 93–99 (1970).

28. Rodríguez-Sáiz, M. *et al.* *Blakeslea trispora* genes for carotene biosynthesis. *Appl. Environ. Microbiol.* **70**, 5589–5594 (2004).

29. Chen, Y. *et al.* Lycopene overproduction in saccharomyces cerevisiae through combining pathway engineering with host engineering. *Microb. Cell Factories* **15**, 113 (2016).

30. Xu, Z., Tian, B., Sun, Z., Lin, J. & Hua, Y. Identification and functional analysis of a phytoene desaturase gene from the extremely radioresistant bacterium *Deinococcus radiodurans*. *Microbiology* **153**, 1642–1652 (2007).

31. Ruiz-Hidalgo, M., Benito, E., Sandmann, G. & Eslava, A. The phytoene dehydrogenase gene of *phycomyces*: regulation of its expression by blue light and vitamin A. *Mol. Gen. Genet. MGG* **253**, 734–744 (1997).

32. Velayos, A., Blasco, J. L., Alvarez, M. I., Iturriaga, E. A. & Eslava, A. P. Blue-light regulation of phytoene dehydrogenase (carB) gene expression in *Mucor circinelloides*. *Planta* **210**, 938–946 (2000).

33. Heider, S. A., Peters-Wendisch, P. & Wendisch, V. F. Carotenoid biosynthesis and overproduction in *Corynebacterium glutamicum*. *BMC Microbiol.* **12**, 198 (2012).

34. Sandmann, G., Woods, W. S. & Tuveson, R. W. Identification of carotenoids in *Erwinia herbicola* and in a transformed *Escherichia coli* strain. *FEMS Microbiol. Lett.* **71**, 77–82 (1990).

35. Guo, W. *et al.* Cloning and characterization of a phytoene dehydrogenase gene from marine yeast *Rhodosporidium diobovatum*. *Antonie van Leeuwenhoek* **107**, 1017–1027 (2015).

36. Moliné, M., Libkind, D. & van Broock, M. Production of torularhodin, torulene, and β-carotene by *Rhodotorula* yeasts. In *Microbial Carotenoids from Fungi*, 275–283 (Springer, 2012).

37. Verdoes, J. C. *et al.* Metabolic engineering of the carotenoid biosynthetic pathway in the yeast *Xanthophyllomyces dendrorhous* (*Phaffia rhodozyma*). *Appl. Environ. Microbiol.* **69**, 3728–3738 (2003).

38. Verdoes, J. C., Misawa, N. & van Ooyen, A. J. Cloning and characterization of the astaxanthin biosynthetic gene encoding phytoene desaturase of *Xanthophyllomyces dendrorhous*. *Biotechnol. Bioeng.* **63**, 750–755 (1999).

39. Jin, J.-M., Lee, J. & Lee, Y.-W. Characterization of carotenoid biosynthetic genes in the ascomycete *Gibberella zeae*. *FEMS Microbiol. Lett.* **302**, 197–202 (2010).

40. Prado-Cabrero, A. *et al.* Deviation of the neurosporaxanthin pathway towards β-carotene biosynthesis in *Fusarium fujikuroi* by a point mutation in the phytoene desaturase gene. *The FEBS J.* **276**, 4582–4597 (2009).

41. Harding, R. W., Huang, P. & Mitchell, H. K. Photochemical studies of the carotenoid biosynthetic pathway in *Neurospora crassa*. *Arch. Biochem. Biophys.* **129**, 696–707 (1969).

42. Hausmann, A. & Sandmann, G. A single five-step desaturase is involved in the carotenoid biosynthesis pathway to β-carotene and torulene in *Neurospora crassa*. *Fungal Genet. Biol.* **30**, 147–153 (2000).

43. Davoli, P., Mierau, V. & Weber, R. Carotenoids and fatty acids in red yeasts *Sporobolomyces roseus* and *Rhodotorula glutinis*. *Appl. Biochem. Microbiol.* **40**, 392–397 (2004).

44. Li, C. *et al.* A single desaturase gene from red yeast *Sporidiobolus pararoseus* is responsible for both four-and five-step dehydrogenation of phytoene. *Gene* **590**, 169–176 (2016).
